# Bottom-hole pressure drawdown management of fractured horizontal wells in shale gas reservoirs using a semi-analytical model

**DOI:** 10.1038/s41598-022-26978-2

**Published:** 2022-12-28

**Authors:** Yingying Xu, Xiangui Liu, Zhiming Hu, Xianggang Duan, Jin Chang

**Affiliations:** 1 University of the Chinese Academy of Sciences, Beijing, 100049 China; 2 Institute of Porous Flow and Fluid Mechanics, Chinese Academy of Sciences, Lang Fang, 065007 China; 3 Research Institute of Petroleum Exploration and Development, PetroChina, Beijing, 100083 China

**Keywords:** Fossil fuels, Natural gas

## Abstract

Due to strong stress sensitivity resulted from unconventional tight formationsit is of practical interest to formulate a reasonable pressure drawdown plan to improve gas extraction recovery. The impact of water-shale interactions on the reservoir permeability was previously ignored in the managed pressure drawdown optimization. The controlled-pressure production dynamic analysis was mostly conducted using numerical simulation, lack of rigorous theoretical support. Hence in this paper, a theoretical production prediction model was proposed and verified with HIS RTA 2015by incorporating multiple pressure drawdown mechanisms and various non-linear gas flow process. The on-site production effects dominated by two different pressure drop methods was further compared, indicating that compared to depressurization production, the production reversion can occur in the controlled pressure production process and the EUR of single well can be increased by about 30% under the control of managed pressure drawdown approach. Finally, the pressure drawdown optimization strategy was carried out on the field test from the both production effect and economic benefits, which demonstrated that the best economic solution can generally be obtained in the early stage of production. The research results can be closely linked to the on-site production practice of shale gas wells, providing insights into designing optimized production strategy scheme.

## Introduction

Since 2010, three industrial production demonstration zones in the marine shale gas reservoirs have been established in China, making China the third country in the world to realize the commercial development of shale gas reservoirs^1^. Deep shale gas resources will become the main body of natural gas production in the future. By 2040, China is likely to be the largest shale gas producing area after North America ^2^. Shale gas has become an important replacement resource to make up for the shortage of conventional energy^2,3^, and it has affected profoundly the world's energy production and consumption patterns.

Shale gas reservoir is typically characterized by low-porosity and extremely low-permeability. The permeability of the reservoir with poor pore connectivity is between 10^–5^ ~ 10^−3^mD^4–6^. The large resistance in gas seepage of shale gas reservoir makes no productivity under natural conditions. The horizontal well and hydraulic fracturing technology are generally adopted to achieve industrialized gas flow. The rapid decline rate of initial daily gas production for shale gas wells can be attributed to the strong stress sensitivity. During the production process of a shale gas well, the reservoir conductive medium is gradually compacted with the increase of the closure pressure, and the shale gas flow channel is deformed, resulting in a decrease in seepage capacity.

In the practice of shale gas reservoir development, it is necessary to clarify a suitable bottom hole pressure drawdown strategy, which is conducive to reducing the daily gas production decline rate and increasing the ultimate productivity of shale gas single well. Generally, the production methos of shale gas wells can be divided into depressurization production and controlled pressure production. Depressurization production means that there is no flow restriction at the wellhead, and the bottom hole flowing pressure at the initial production stage of gas well is rapidly reduced to the constant pressure. The shale gas wells with depressurization production can show high initial productivity and rapid decline with daily gas production, resulting in short or even nonexistent stable production period. Improper control of the flowback rate is likely to cause proppant backflow, reduce fracture conductivity and even cause reservoir damage. While pressure-controlled production generally restricts gas flow by adjusting different nozzles sizes at the wellhead to delay the decline rate of wellhead pressure, and achieve long-term stable production at the expense of initial high production. The shale gas development practice shows that the pressure-controlled production is better to increase the EUR per well by 28~30% than the depressurization production^7–10^.

Formulating a reasonable pressure-controlled production plan is the key to obtaining ideal productivity of over-pressured fractured shale gas reservoirs. The common research methods for the dynamic performance analysis of shale gas wells with pressure drawdown management mainly includes: field test^11^, physical simulation^12–14^, and numerical simulation^11,15,16^. Restricted to the strong regionality and high research costs, field tests are mostly used in large-scale field tests to provide engineering practice knowledge for theoretical scientific research and physical simulation work. The physical simulation method provides sufficient experimental support and numerical parameters for the controlled pressure production theory. Meanwhile, a series of core-scaled control pressure development experiments cannot accurately reflect the gas flow process in the kilometer-scale shale reservoir. The above-mentioned numerical model simplifies the pressure control mechanism and the gas flow mechanism in the reservoir, which is inconsistent with the complex production process of on-site shale gas reservoirs.

The pressure-controlled production simulation based on the comprehensive pressure-controlled mechanism is scientific and theoretical to the optimization of shale gas reservoir production strategies. Currently, the four common industrialized and recognized pressure-controlled mechanisms include (1) artificial fracture conductivity loss^17–19^; (2) micro-fracture stress sensitivity^16,20,21^; (3) matrix stress sensitivity^22,23^; (4) proppant reflux theory^24,25^. So far, the pressure control mechanism analysis is mainly conducted by numerical simulation, which lacks the support and verification of experimental research and theoretical models. In addition, the current pressure-control mechanisms mostly focus on the stress-sensitivity of gas-saturated rock, and the impact of water–rock interaction on reservoir damage has not been considered. Literature studies^26,27^ explained that much fracturing fluids in long-term contact with the formation causes the particles to fall off the reservoir and the clay-swelling, leading to the enhancement in the reservoir stress sensitivity. Thus, the water–rock interactions cannot be ignored in the research on pressure control mechanisms of shale gas wells.

The aim of this work is to establish the theoretical production prediction model for managed pressure drawdown not considering the proppant backflow, but focusing on the elastic embedding and deformation of the proppant-fractures, the hydration in equivalent fracture network area and the matrix stress sensitivity, coupled with the various nonlinear gas flow effects, which was validated with the numerical simulator IHS RTA2015. Furthermore, the comparative analysis of on-site production effects between managed pressure drawdown and high pressure drawdown was carried out, and the necessity of managed pressure production for deep reservoirs was clarified. Finally, the optimization of pressure drawdown management strategy was conducted from the perspective of production effects and economic benefits, which has scientific and theoretical reference for guiding the formulation of on-site shale gas well production plans, and maximizes the single well EUR and recovery efficiency of gas wells.

## Pressure control mechanism

### Artificial fracture conductivity loss

**Figure 1:**
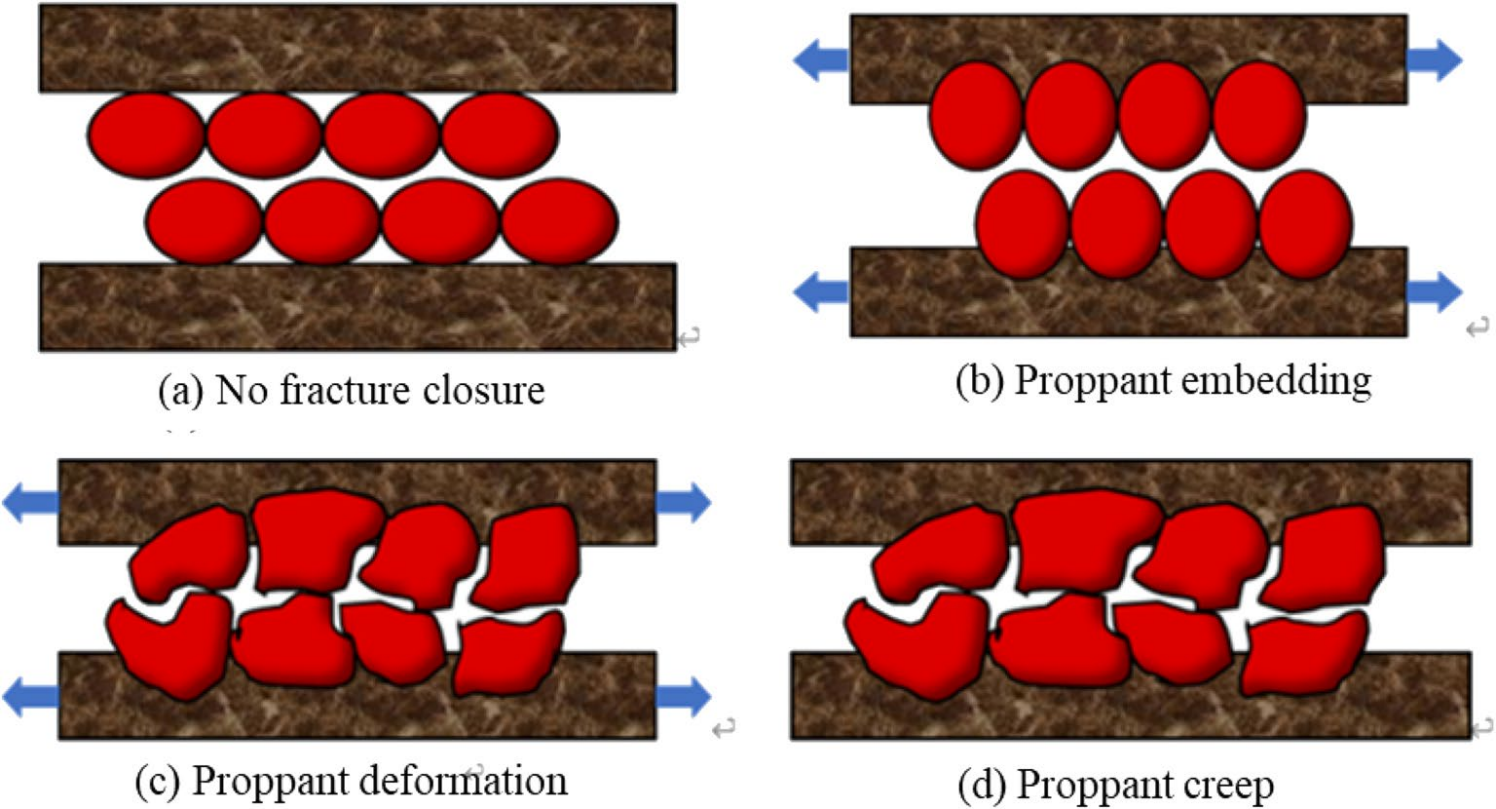
Schematic diagram of crack width change^9^.

The effective support performance of proppants to fractures is the key to forming the required fracture conductivity^28^. The increase in the effective net stress pressure causes the proppant inside the artificial fracture to be embedded, deformed and ruptured (Fig. 1), thus the effective flow channel width of the fracture is reduced, and the fracture conductivity can be impaired^29^. Pressure-controlled production can appropriately adjust the bottom hole pressure decline rate, control the increase in effective stress, and effectively avoid the reduction of fracture conductivity, to increase the long-term EUR of the gas well. Assuming that the impact of proppant elastic embedding and deformation of proppant on the width of hydraulic fractures is linearly related to the net confining pressure^18^, the expressions (1) and (2) are expressed as follows:

Width loss caused by elastic embedding of proppant:

1 $$\Delta w_{E} = 2\Delta p_{avg} (1 - v_{m}^{2} )D_{p} /2/E_{p}$$

Width loss caused by elastic fracture of proppant:

2 $$\Delta w_{D} = 1.04\cdot \ D_{p} \cdot \Delta p_{avg} (1 - v_{P}^{2} )/E_{p}$$

The effective width of the proppant-fracture is:

3 $$w_{e} = w_{0} - \Delta w_{E} - \Delta w_{D}$$

where $p_{avg}$ is average pore pressure, Pa; $v_{m}$ is reservoir Poisson's ratio, dimensionless; $D_{p}$ is median value of proppant particle size, m; $E_{p}$ is Young’s elastic modulus of proppant, Pa; w_0_ is initial width of hydraulic fracture, m; $v_{p}$ is proppant Poisson's ratio, dimensionless.

### Microfracture water–rock interaction

**Figure 2:**
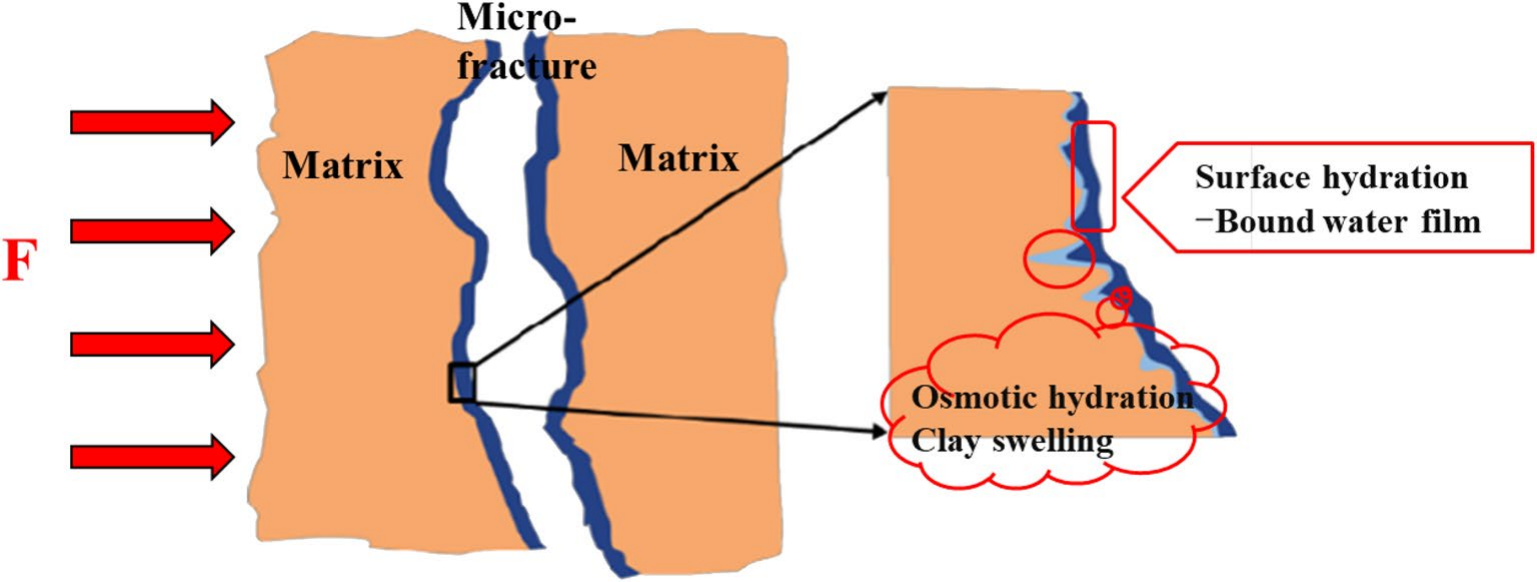
Schematic diagram of water–rock interaction (reservoir scale).

**Figure 3:**
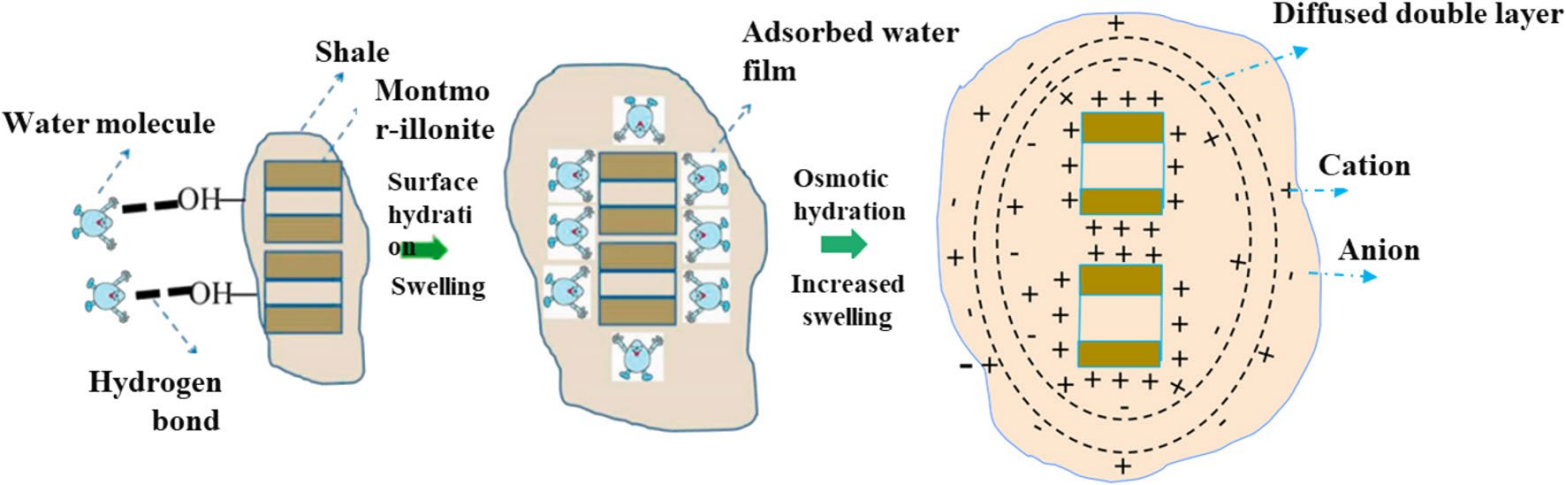
Schematic diagram of rock hydration mechanism (micro-scale).

Compared with matrix and proppant hydraulic fractures, unsupported microfractures are more sensitive to net reservoir stress. The invasion of excess fracturing fluid into the fractures near the well hole bore can make the fractures surface hydration^30,31^ and osmotic hydration^30,31^, successively causing clay minerals to expand^32^, weakening the mechanical properties of the reservoir^33^, and enhancing the stress sensitivity (Figs. 2 and 3). Even after the stress is restored, the permeability can only recover to 10% to 15% of the initial permeability^34^. In this work, the permeability is assumed to decrease exponentially with the increase of effective stress^35^. The stress sensitivity coefficient after fracturing fluid soaking is not constant, but is negatively correlated with effective stress^9^.

The permeability in the water-bearing equivalent fracture network is:

4 $$K_{f} = K_{fi} e^{{ - \gamma_{f} (p_{e} - p_{f} )}}$$

where:

5 $$\gamma_{f} = a(p_{e} - p_{f} ) + \gamma_{f0}$$

where $K_{f}$ is facture permeability considering stress sensitivity, m^2^; $K_{fi}$ is intrinsic fracture permeability, m^2^; $\gamma_{f}$ is stress sensitivity coefficient of the fracture, Pa^-1^; $p_{e}$ is initial formation pressure, Pa; $p_{f}$ is fracture pore pressure, Pa.

### Matrix stress sensitivity

**Figure 4:**
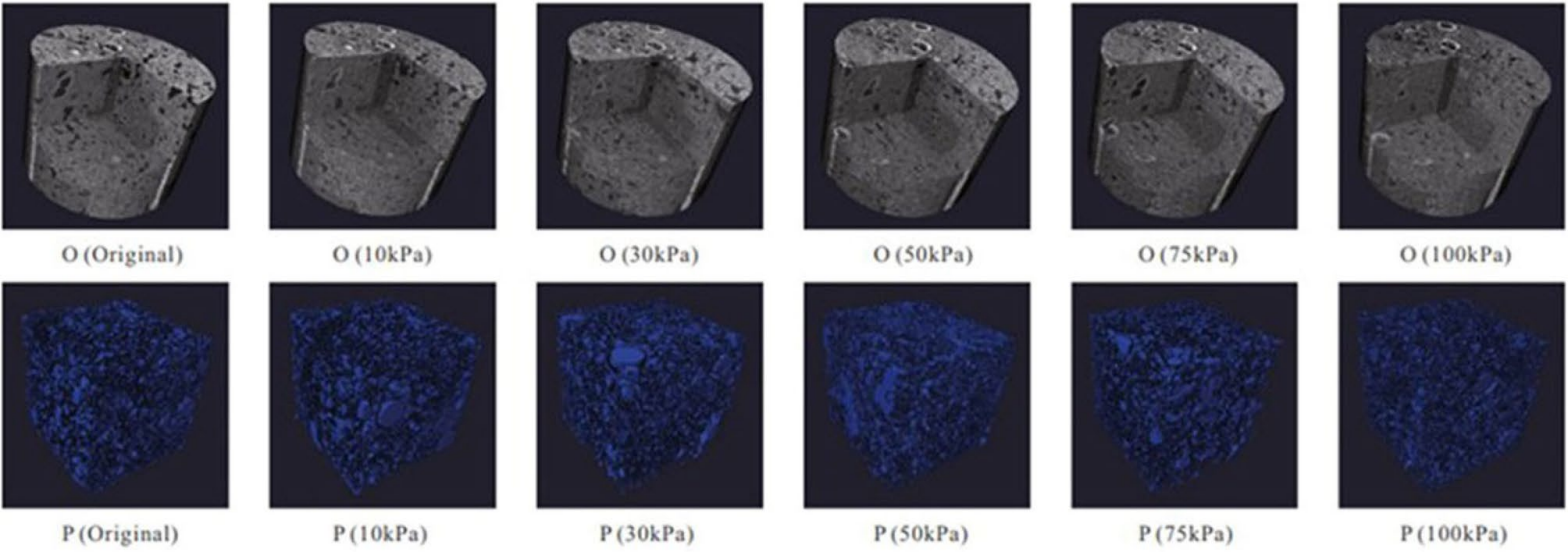
Changes of rock skeleton and pore structure with confining pressure^22^.

The increase effective stress on the rock skeleton of the reservoir results in obvious elastoplastic deformation of the rock pore structure (Fig. 4), and thus matrix permeability, porosity, and rock physical parameters have changed. Proper pressure-controlled production can reduce the shrinkage rate of rock pore volume and improve the connectivity between effective pores. Therefore, an exponential stress sensitivity empirical model^35^ was introduced in the paper to characterize the influence of matrix stress sensitivity on matrix seepage capacity:

6 $$K_{m} = K_{mi} e^{{ - \gamma_{m} (P_{e} - P_{m} )}}$$

where $K_{m}$ is matrix permeability considering stress sensitivity, m^2^; $K_{mi}$ is intrinsic matrix permeability, m^2^; $\gamma_{m}$ is stress sensitivity coefficient of the fracture, Pa^-1^; $p_{m}$ is matrix pore pressure, Pa.

## Methodology

### Physical model description

**Figure 5:**
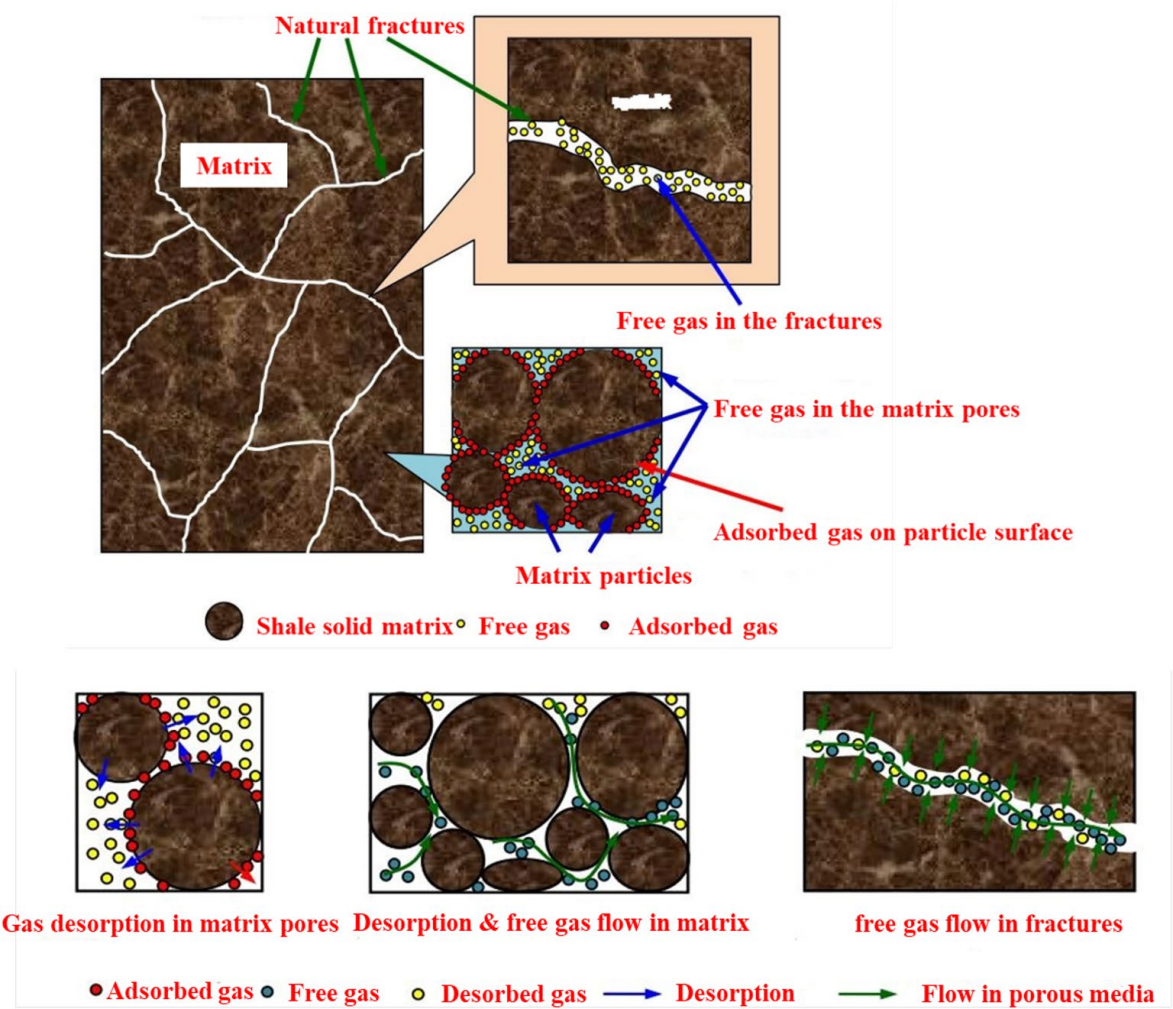
Mass transfer process of shale reservoir gas to the wellbore.

The diversity of pore structure scales in shale reservoirs makes the shale gas flow process complicated, and the flow path covers the molecular scale to the macro scale^36^. The shale gas production period is generally attributed to multiple migration mechanisms across scales, and it is mainly divided into three stages (Fig. 5): In the first stage, free gas in the proppant fractures and unproppant fractures moves towards the wellbore by pressure drop near the wellbore; During the second stage, free gas in the matrix flow to natural fractures, motivated by the pressure difference between the matrix and the fracture; In the third stage, the reservoir pressure change in the matrix pores can promote microscopic gas flow such as the diffusion and slippage of free gas and the surface diffusion of adsorbed gas to enhance gas flow capacity in the matrix. When the matrix pore pressure is declined to the critical desorption pressure, the adsorbed gas starts to mobilize into free gas in the matrix pores.

**Figure 6:**
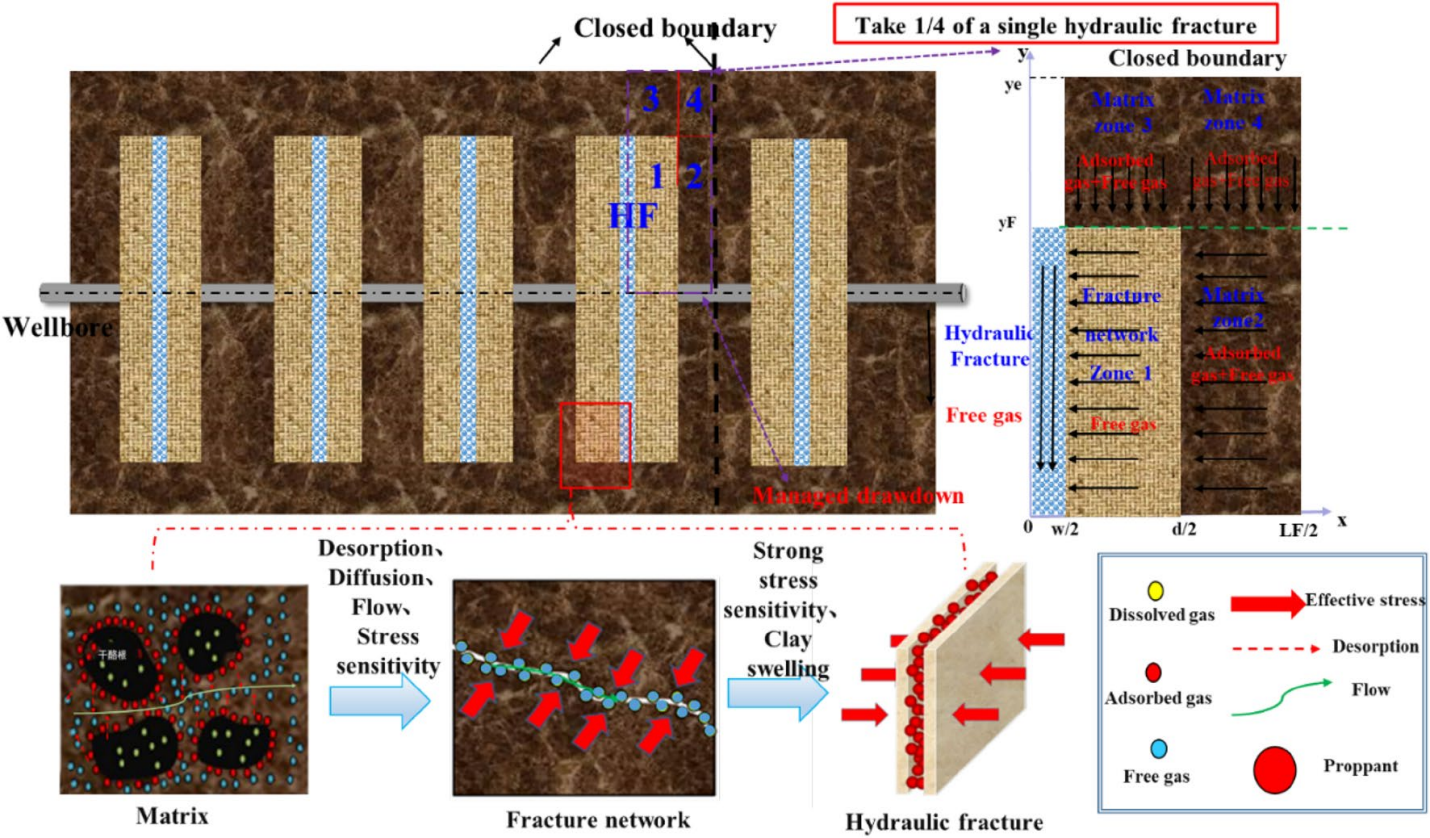
Simplified five-zone composite physical model of multi-fractured horizontal well.

Irregular fractures distribution and strong heterogeneity in the fractured reservoir make it difficult to acquire the specific fracture and certain reservoir parameters. Thus, the fracture and matrix can be simplified to facilitate the calculation process. It is assumed that all the hydraulic fractures can be characterized by bi wing transverse^37^, note that the increased effective stress may lead to elastic embedding and deformation of proppant in hydraulic fractures. Furthermore, the secondary fracture network and matrix are considered as equivalent medium. Considering that there are some unstimulated reservoir zones between the hydraulic fractures and outside the fracture tip, the proposed model in the paper can include the following five flow areas: hydraulic fractures, fracture network area 1, matrix area 2 between hydraulic fractures, and unstimulated matrix area 3 and matrix area 4, as is shown in the Fig. 6. The single-phase methane gas flow behavior in different areas is one-dimensional flow. The stress sensitivity, high-pressure adsorption, diffusion and viscous flow are all considered in the matrix 2, 3, and 4. Moreover, the hydration expansion of unproppant fracture network can aggravate the clay swelling, which is the focus of pressure control protection for gas wells.

Where $\psi_{e}$ is initial formation pseudo-pressure, Pa^2^/(Pa $\bullet$ s); $\psi$ is the formation pseudo-pressure, Pa2/(Pa $\bullet$ s); $K_{F}$ is hydraulic fracture permeability, m^2^; $\varphi_{2}$ is porosity of the matrix zone 2, ,dimensionless; $\varphi_{1}$ is porosity of fracture network, dimensionless; $\varphi_{3}$ is porosity of matrix zone 3. m^2^; $C_{t3}$ is comprehensive compressibility of matrix zone 3, Pa^-1^; $C_{t2}$ is comprehensive compressibility of matrix zone 2, Pa^-1^; $C_{t1}$ is comprehensive compressibility of fracture network, Pa^-1^; $t_{a}$ is pseudo-time.s; $L_{f}$ is hydraulic fracture spacing, m; $A_{cw}$ is wellbore crossflow area,m^2^; $\varphi_{F}$ is porosity of hydraulic fracture, m; $C_{tF}$ is comprehensive compressibility of hydraulic fracture,Pa^-1^; $K_{3a}$ is apparent permeability of matrix zone 3,m^2^; $K_{1}$ is fracture network permeability,m^2^;$K_{2}$ is matrix zone 2 permeability,m^2^;$d$ is the width of stimulated reservoir zone, m.

### Mathematical model

#### Governing equation for gas flow in matrix of zone 4

The matrix gas of zone 4 merges into the matrix of zone 2 along the y direction. Considering the stress sensitivity, the supercritical desorption, the diffusion and viscous flow, the outer boundary condition is closed and the pressure on the inner boundary is continuous. the gas percolation equation in the matrix of zone 4 can be derived in Eq. (7):

7 $$\, \varphi_{{4}}^{{}} C_{{t{4}}} \frac{{\partial P_{{4}} }}{\partial t}{ = }\frac{\partial }{\partial y}\left( {\rho_{{\text{g}}} \frac{{K_{{{4}a}} }}{{\mu_{{4}} }}\frac{{\partial P_{{4}} }}{\partial y}} \right)$$

where $\varphi_{{4}}^{{}}$ is porosity of matrix zone 4, dimensionless; $C_{{t{4}}}$ is the matrix comprehensive compressibility coefficient, Pa^-1^; $P_{{4}}$ is pore pressure in matrix zone 4, Pa; $t$ is production duration, s; $\rho_{{\text{g}}}$ is gas density,,kg/m^3^; $K_{{{4}a}}$ is apparent permeability of matrix zone 4, m^2^; $\mu_{{4}}$ is gas viscosity, $Pa\bullet s$.

where: the matrix comprehensive compression factor is:

8 $$C_{{{\text{t4}}}} = C_{{{\text{g4}}}} + C_{{{\text{d4}}}} { + }C_{{{\text{f4}}}}$$

Gas compression factor:

9 $$C_{{{\text{g4}}}} = \frac{1}{{\rho_{{\text{g}}} }}\frac{{\partial \rho_{{\text{g}}} }}{{\partial P_{{4}} }}$$

where $C_{{{\text{g4}}}}$ is gas compressibility, Pa^−1^; $C_{{{\text{d4}}}}$ is modified supercritical desorption gas compression coefficient of matrix zone 4, Pa^−1^; $C_{{{\text{f4}}}}$ is formation compressibility, Pa^−1^.

**Figure 7:**
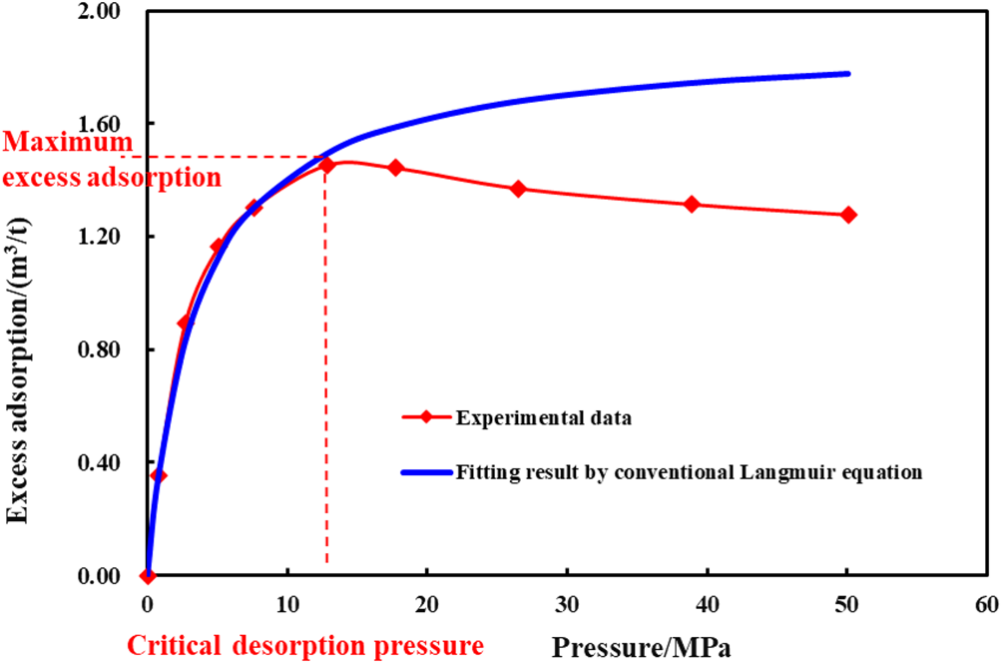
High pressure isotherm adsorption curve for shale^38^.

Considering that the conventional Langmuir equation cannot be applied to high pressure isotherm adsorption process (Fig. 7), the high pressure isotherm adsorption model^38^ is adopted and desorption gas compression factor^39^ is transformed as Eq. (10):

10 $$C_{{{\text{d4}}}} = V_{L} \frac{{(1 - \varphi_{{4}} )TZP_{{{\text{sc}}}} }}{{\varphi_{{4}} Z_{{{\text{sc}}}} T_{{{\text{sc}}}} }}\frac{{P_{L} }}{{P_{{4}} (P_{{4}} + P_{L} )^{2} }}(1 - \frac{{\rho_{{\text{g}}} }}{{\rho_{{\text{a}}} }})$$

where $V_{L}$ is Langmuir volume, m^3^/m^3^; $\varphi_{{4}}$ is porosity of the matrix zone 4, dimensionless; $T$ is formation temperature, K; Z is gas compression factor, dimensionless; $P_{{{\text{sc}}}}$ is standard atmospheric pressure, Pa; $Z_{{{\text{sc}}}}$ is standard gas compression factor, taking the value of 1, dimensionless; $T_{{{\text{sc}}}}$ is standard atmospheric temperature, K; $P_{L}$ is Langmuir pressure, Pa; $\rho_{{\text{a}}}$ is absorbed gas density, kg/m^3^.

In this paper, the stress sensitivity of the matrix and the desorption of gas in the matrix are coupled, and the apparent permeability model of gas flow in the shale matrix micro/nanopores based on molecular dynamics theory was used to superimpose the viscous flow and Knudsen diffusion of real gas molecules with weighting coefficients:

11 $$K_{4a} = \left( {\frac{1}{{K_{ne} + 1}}K_{4i} + \frac{{K_{ne} }}{{K_{ne} + 1}}C_{g} D\mu } \right) \bullet \left( {1 + \frac{{C_{d4} }}{{C_{d4} + C_{g4} + C_{f4} }}} \right)$$

where:

effective Knudsen number:

12 $$K_{ne} = \frac{\lambda }{{r_{e} }} = \frac{{K_{B} T}}{{r_{e} \sqrt 2 \pi d^{2} \overline{p} }}$$

Considering stress sensitivity and desorption process the effective hydraulic flow radius:

13 $$r_{e} = r_{0} e^{{ - \gamma_{m} (p_{e} - p_{m} )/2}} - \theta d_{{CH_{4} }}$$

Surface coverage of adsorbed gas on matrix pore wall:

14 $$\theta = \frac{{p_{4} }}{{p_{4} + p_{L} }}(1 - \frac{{\rho_{g} }}{{\rho_{a} }})$$

where $K_{4a}$ is apparent permeability of matrix zone 4, m^2^; $K_{4i}$ is intrinsic permeability of matrix zone 4, m^2^; $D$ is gas diffusion coefficient, m^2^/s; $\lambda$ is molecular free path of methane molecule, m; $r_{e}$ is effective hydraulic radius, m; $r_{0}$ is original hydraulic radius, m; $K_{B}$ is Boltzmann constant, 1.38065*10–23 J/K; $d$ is methane molecular collision diameter,m; $\overline{p}$ is average pore pressure in the circular pore, m; $d_{{CH_{4} }}$ is methane molecular collision diameter, m.

The pseudo pressure and pseudo-time^37^are adopted to linearize the high-pressure physical property parameters in the flowing control equation to solve the equations easily.

The pseudo-pressure is:

15 $$\psi_{{}} = \int\limits_{0}^{P} {\frac{2P}{{\mu Z}}dP_{{}} }$$

The pseudo-time is:

16 $$t_{a} = \int\limits_{0}^{t} {\frac{{\mu_{i} C_{ti} }}{{\mu (P)C_{t} (P)}}} dt$$

where $\mu_{i}$ is gas viscosity at initial formation pressure condition, $Pa\bullet s$; $C_{ti}$ is comprehensive compression factor of matrix zone 4 at initial formation pressure condition, Pa^-1^; $\mu (p)$ is gas viscosity at formation pressure of $p$, $Pa\bullet s$; $C_{ti}$ is comprehensive compression factor of matrix zone 4 at formation pressure of $p$,, Pa^-1^;

According to the definition of pseudo-pressure and pseudo-time and the dimensionless parameters definition Table 1, Eq. (7) can be rewritten as follows:

17 $$\frac{{\partial \psi_{4D} }}{{\partial t_{a} }} = \frac{1}{{\eta_{4D} }}\frac{{\partial^{2} \psi_{4D} }}{{\partial y^{2} }}$$

**Table 1:** The definition of dimensionless parameters.

Dimensionless parameter	Definition	Dimensionless parameter	Definition
Dimensionless pseudo-pressure	$\psi_{D} = \frac{{\psi_{e} - \psi }}{{\psi_{e} - \psi_{wf} }}$	Dimensionless production	$\frac{1}{{q_{D} }} = \frac{{T_{sc} K_{F} \sqrt {A_{cw} } (\psi_{e} - \psi_{wf} )}}{{p_{sc} q_{sc} T}}$
Dimensionless pseudo-time	$t_{aD} = \frac{{K_{F} t_{a} }}{{\mu (\varphi_{1} C_{t1} + \varphi_{2} C_{t2} + \varphi_{3} C_{t3} )A_{cw} }}$	Storage capacity ratio	$w_{i} = \frac{{\varphi_{i} C_{ti} }}{{\varphi_{1} C_{t1} + \varphi_{2} C_{t2} + \varphi_{3} C_{t3} }}(i = 1,2,3)$
Dimensionless length in x direction	$x_{D} = \frac{2x}{{L_{f} }}$	Zone2-4Mass transfer coefficient	$\lambda_{24} = \frac{{12K_{4} }}{{L_{F}^{2} K_{2} }}A_{cw}$
Dimensionless length in y direction	$y_{D} = \frac{y}{{\sqrt {A_{cw} } }}$	Zone3-1mass transfer coefficient	$\lambda_{13} = \frac{{12K_{1} }}{{L_{F}^{2} K_{3} }}A_{cw}$
Dimensionless conductivity in zone 3	$\eta_{3D} = \frac{{K_{F} }}{{\varphi_{1} C_{t1} + \varphi_{2} C_{t2} + \varphi_{F} C_{tF} }}\frac{{\varphi_{3} C_{t3} }}{{K_{3a} }}$	Zone1-Fmass transfer coefficient	$\lambda_{1F} = \frac{{12K_{1} }}{{L_{F}^{2} K_{F} }}A_{cw}$
Dimensionless conductivity in zone 4	$\eta_{4D} = \frac{{K_{F} }}{{\varphi_{1} C_{t1} + \varphi_{2} C_{t2} + \varphi_{F} C_{tF} }}\frac{{\varphi_{4} C_{t4} }}{{K_{4a} }}$	Dimensionless formation conductivity	$R_{CD} = \frac{{K_{1} d}}{{K_{2} L_{F} }}$

Boundary conditions can be expressed in dimensionless form are as follows:

18 $$\psi_{4D} (y_{D} ,0) = 0$$

19 $$\psi_{4D} (y_{FD} ,t_{aD} ) = \psi_{2D}$$

20 $$\left. {\frac{{\partial \psi_{4D} (y_{D} ,t_{aD} )}}{{\partial y_{D} }}} \right|_{{y_{D} = y_{{{\text{eD}}}} }} = 0$$

#### Governing equation for gas flow in the matrix of zone 3

Considering the reservoir stress sensitivity, gas desorption, diffusion and viscous flow from matrix zone 3 into zone 1 along the y direction, the dimensionless gas percolation equation in the matrix of zone 3 can be derived in Eq. (21):

21 $$\frac{{\partial \psi_{3D} }}{{\partial t_{aD} }} = \frac{1}{{\eta_{3D} }}\frac{{\partial^{2} \psi_{3D} }}{{\partial y_{D}^{2} }}$$

As the outer boundary condition is closed and the pressure on the inner boundary is continuous, the dimensionless initial and boundary conditions are reflected in Eqs. (22–24) as follows:

22 $$\psi_{3D} (y_{D} ,0) = 0$$

23 $$\psi_{3D} (y_{FD} ,t_{aD} ) = \psi_{1D}$$

24 $$\left. {\frac{{\partial \psi_{3D} (y_{D} ,t_{aD} )}}{{\partial y_{D} }}} \right|_{{y_{D} = y_{{{\text{eD}}}} }} = 0$$

#### Governing equation for gas flow in matrix of zone 2^40^

Considering the unsteady gas flow exchange in matrix zone 4 and matrix zone 2, the dimensionless gas seepage equation in the matrix of zone 2 is established in Eq. (25):

25 $$\frac{{\partial^{2} \psi_{2D} }}{{\partial x_{D}^{2} }} = \frac{{3w_{2} }}{{\lambda_{12} }}\frac{{\partial \psi_{2D} }}{{\partial t_{aD} }} - \left. {\frac{6}{{\lambda_{24} y_{FD} }}\frac{{\partial \psi_{4D} }}{{\partial y_{D} }}} \right|_{{y = y_{FD} }}$$

The outer boundary is closed, the inner boundary pressure is continuous, the initial and boundary conditions are expressed in Eqs. (26–28):

The initial condition:

26 $$\psi_{2D} (x_{D} ,0) = 0$$

Outer boundary:

27 $$\left. {\frac{{\partial \psi_{2D} (x_{D} ,t_{aD} )}}{{\partial x_{D} }}} \right|_{{x_{D} = 1}} = 0$$

Inner boundary:

28 $$\psi_{2D} (x_{d} ,t_{aD} ) = \psi_{1D}$$

#### Governing equation for gas flow in fracture network of zone 1

Consider the influence of hydration on the seepage capacity of the equivalent fracture network in zone 1, the flow rate on the outer boundary between zone 1 and zone 2 is continuous, and the inner boundary pressure is continuous. The dimensionless gas flow equation in fracture network zone 1 can be depicted in expression (29):

29 $$\frac{{\partial^{2} \psi_{1D} }}{{\partial x_{D}^{2} }} = \frac{{3w_{1} }}{{\lambda_{1F} }}\frac{{\partial \psi_{1D} }}{{\partial t_{aD} }} - \left. {\frac{6}{{\lambda_{13} y_{FD} }}\frac{{\partial \psi_{3D} }}{{\partial y_{D} }}} \right|_{{y = y_{FD} }}$$

The boundary conditions are as followed in Eqs. (30–32):

Initial condition:

30 $$\psi_{1D} (x_{D} ,0) = 0$$

Outer boundary:

31 $$\psi_{1D} (0,t_{aD} ) = \psi_{FD}$$

Inner boundary:

32 $$\left. {\frac{{\partial \psi_{1D} (x_{D} ,t_{aD} )}}{{\partial x_{D} }}} \right|_{{x_{D} = \frac{{d_{D} }}{2}}} = \frac{{\lambda_{12} }}{{\lambda_{1F} }}\left. {\frac{{\partial \psi_{2D} (x_{D} ,t_{aD} )}}{{\partial x_{D} }}} \right|_{{x_{D} = \frac{{d_{D} }}{2}}}$$

#### Governing equation for gas flow in inner zone hydraulic fracture

The gas mass transfer in the hydraulic fracture is mainly dominated by viscous flow, and the elastic embedding and deformation of proppant in the hydraulic fractures cannot be ignored. The dimensionless flow control Eq. (33) in the hydraulic fracture is established:

33 $$\frac{{\partial^{2} \psi_{FD} }}{{\partial y_{D}^{2} }} = w_{F} \frac{{\partial \psi_{FD} }}{{\partial t_{aD} }} - \left. {\frac{{\lambda_{1F} }}{3}\frac{{\partial \psi_{1D} }}{{\partial x_{D} }}} \right|_{{x_{D} = \frac{{d_{D} }}{2}}}$$

where $w_{F}$ is hydraulic fracture width,m.Considering the constant bottom hole pressure of the gas well and the outer boundary is closed, the initial and boundary conditions are as followed:

The initial condition:

34 $$\psi_{FD} (y_{D} ,0) = 0$$

Outer boundary:

35 $$\left. {\frac{{\partial \psi_{FD} (y_{D} ,t_{aD} )}}{{\partial t_{aD} }}} \right|_{{y_{D} = y_{FD} }} = 0$$

Inner boundary:

36 $$\psi_{FD} (0,t_{aD} ) = \psi_{wfD}$$

### Model solution

Dimensionless bottom hole pseudo pressure gradually decreases from the initial reservoir pressure to the constant flowing pressure and then keep constant pressure production, thus the dimensionless bottom hole pressure can be expressed with the dimensionless time as Piecewise function^41^:

37 $$\psi_{wfD} = \frac{{\psi_{e} - \psi_{wf} }}{{\psi_{e} - \psi_{w} }} = \left\{ \begin{gathered} F_{D} (t_{aD} ,t_{BD} )(t_{aD} \le t_{BD} ) \hfill \\ 1(t_{aD} \ge t_{BD} ) \hfill \\ \end{gathered} \right.$$

The Heaviside (x) function^42^ was introduced to transform Eq. (37) into a continuity function (38):

38 $$\psi_{wfD} (t_{aD} ) = F_{D} (t_{aD} ,t_{BD} ) - H(t_{aD} ,t_{BD} )(F_{D} (t_{aD} ,t_{BD} ) - 1)$$

The dimensionless bottom hole pseudo pressure $\psi_{wfD} (t_{aD} )$ was transformed in the Laplace space as the expression (39) :

39 $$\psi_{wfD} (s) = \int_{0}^{{t_{BD} }} {F_{D} } (t_{aD} ,t_{BD} )e^{{ - st_{aD} }} dt_{aD} + \int_{{t_{BD} }}^{\infty } {e^{{ - st_{D} }} } dt_{aD}$$

The second integral term on the right side of the above formula (39) can be simplified to obtain expression (40):

40 $$\psi_{wfD} (s) = \int_{0}^{{t_{BD} }} {F_{D} } (t_{aD} ,t_{BD} )e^{{ - st_{D} }} dt_{aD} + \frac{{e^{{ - st_{BD} }} }}{s}$$

**Figure 8:**
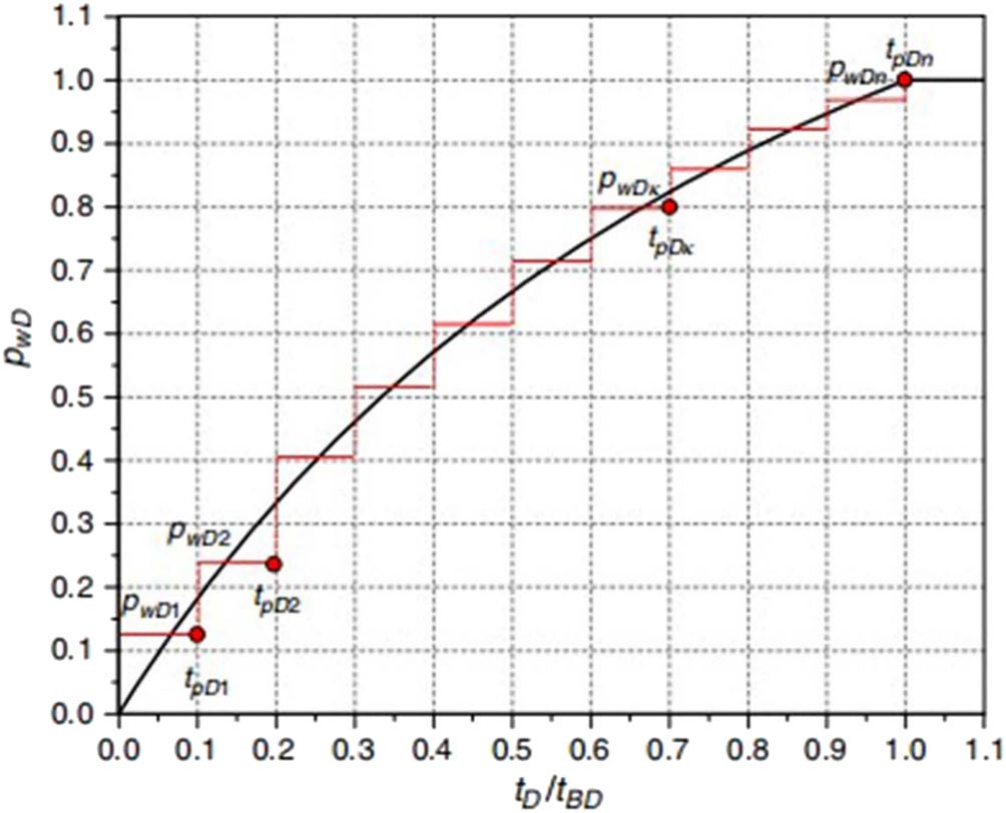
Stepwise schematic of dimensionless BHP drawdown^41^.

Discretize Eq. (40), as shown in Fig. 8:

41 $$\psi_{wfD} (s) = \sum\limits_{k = 1}^{N} {(F_{DK - 1} \cdot } \frac{{e^{{ - st_{aDk - 1} }} - e^{{ - st_{aDk} }} }}{s}) + \frac{{e^{{ - st_{aD} }} }}{s}$$

where:

$$F_{Dk - 1} = 0.5(F_{D} (t_{aDk - 1} ,t_{BD} ) + F_{D} (t_{aDk} ,t_{BD} ))$$

**Figure 9:**
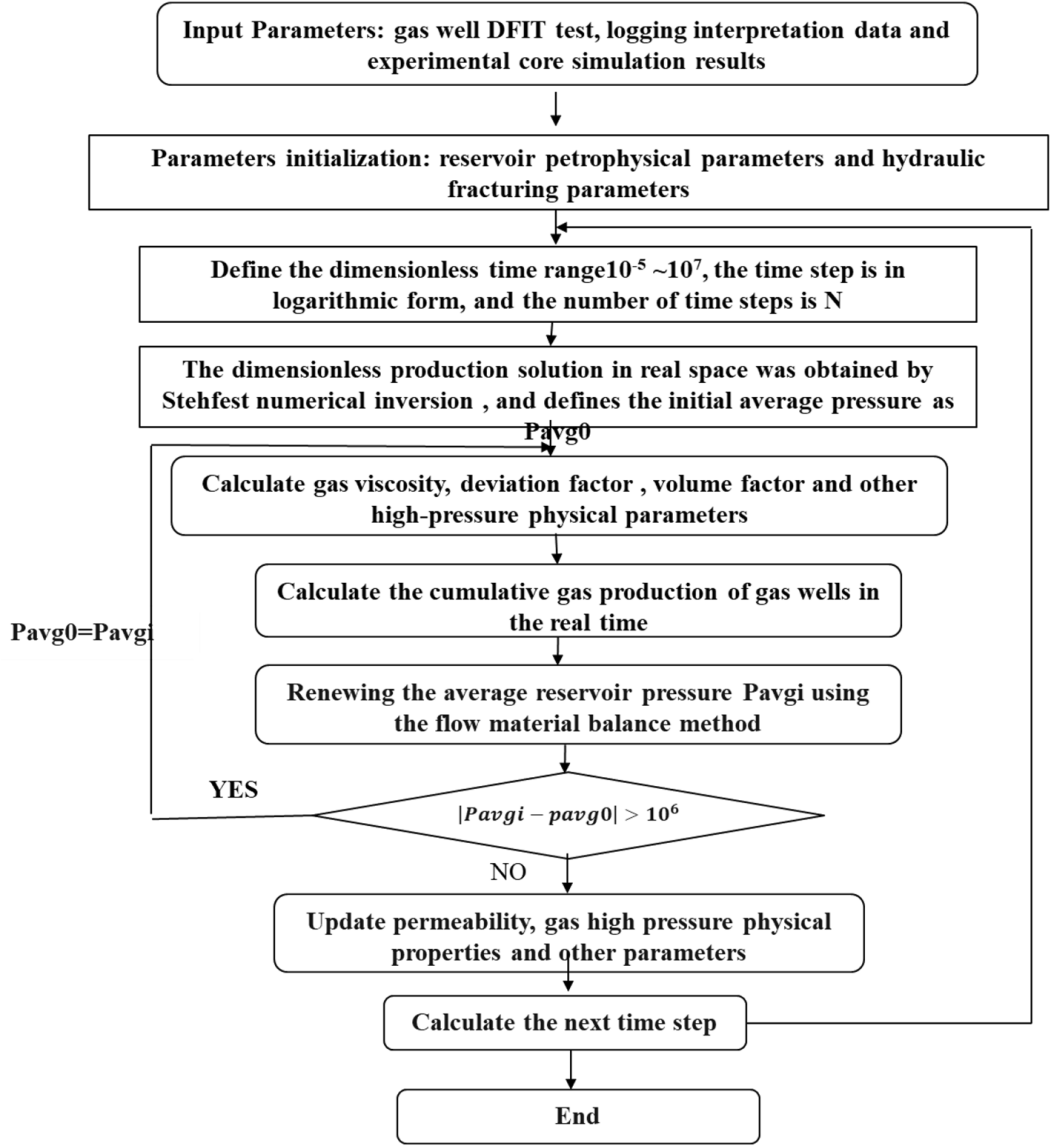
Flow chart of solving pressure control production model.

Next, the dimensionless seepage equations can be transformed in Laplace domain and the final solution was acquired. Then the semi-analytical solution of the dimensionless production in the real space is acquired with the Stehfest numerical inversion^43^. By the Newton iteration method, the production solution in the real space at constant pressure can be derived and the specific solution process was shown in the Fig. 9. As the high lights of this paper are theoretical analysis of dynamic production performance of gas wells with managed pressure drawdown and optimization of pressure drop strategies rather than model derivation, the solution of the model is directly given in this paper. The specific solution derivation details have been illustrated^44^.

The total dimensionless production rate at the bottom hole of the shale gas well in Laplace space can be derived in Eq. (42):

42 $${\text{q}}_{LD} = - \frac{N}{2\pi }\frac{{\partial \psi_{LFD} }}{{\partial y_{D} }}\left| {_{{y_{D} = 0}}^{{}} } \right.$$

where N is the number of hydraulic fractures, dimensionless.

## Model verification and analysis

**Figure 10:**
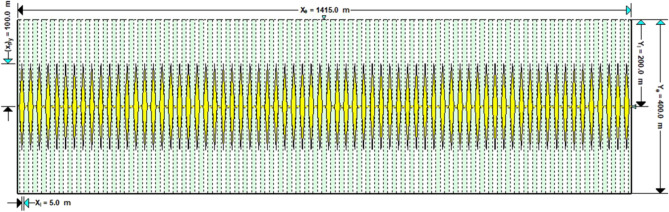
the horizontal multi frac-enhanced fracture region schematic^45^.

**Figure 11:**
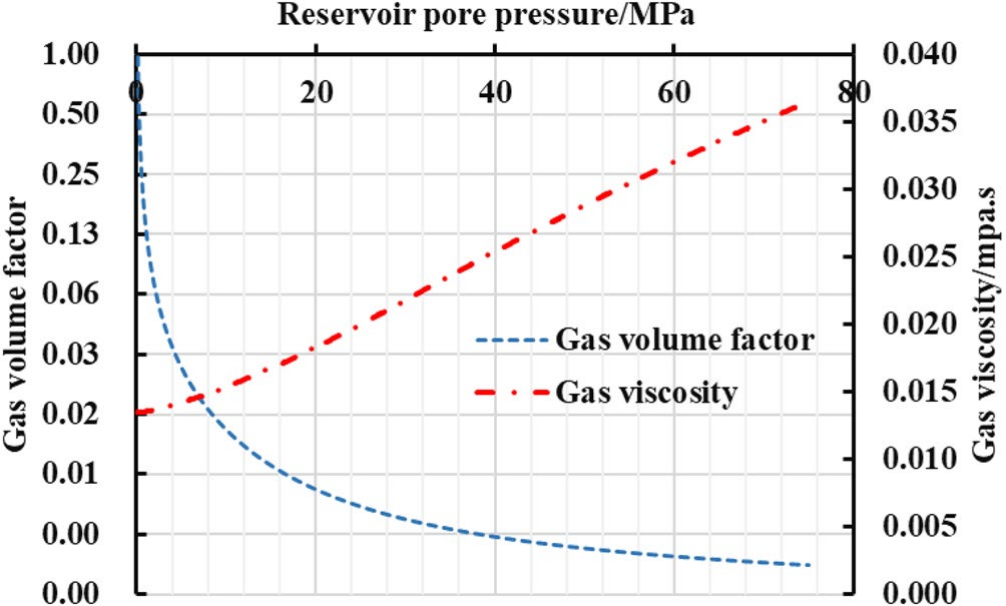
The relationship between gas volume coefficient and viscosity and pressure.

**Figure 12:**
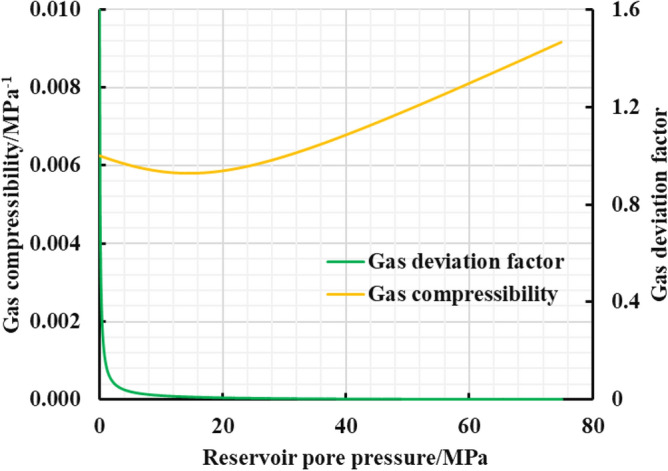
The relationship between gas compressibility and deviation factor and pressure.

**Figure 13:**
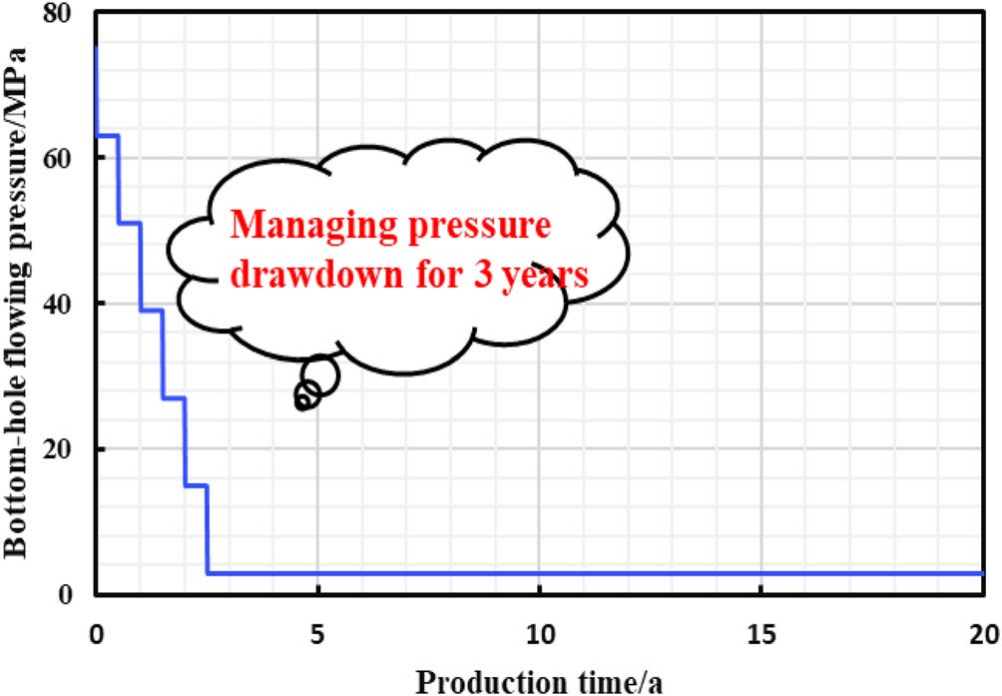
Bottom hole pressure drop path diagram.

**Figure 14:**
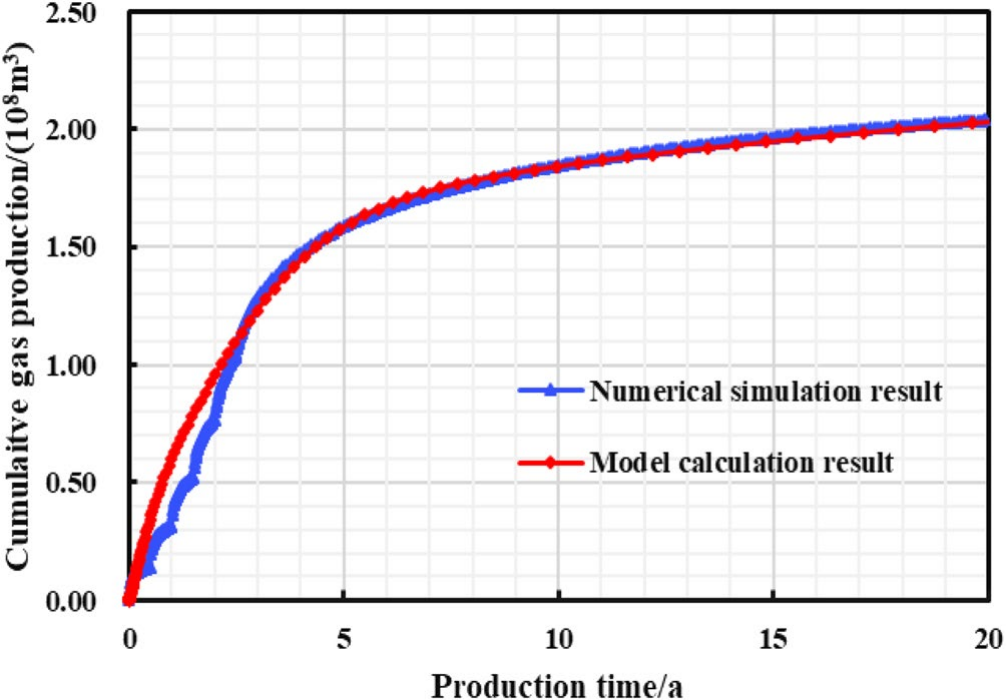
Model verification results.

As is shown in the Fig. 10, a multi-zone composite linear flow numerical model by the IHS simulator was adopted to verify the proposed model with the same model input parameters in the Table 2. The proposed productivity model was simplified due to that the only Darcy flow is considered in the simulator, mainly ignoring the non-linear gas flow mechanism, supercritical desorption and reservoir stress sensitivity, while constant stress sensitivity of the matrix and fracture, high-pressure physical properties (see Figs. 11 and 12), and Langmuir desorption were all considered. The bottom hole pressure drop path in the numerical simulation is assumed to be stepped and the pressure control period is 3 years (Fig. 13), the production prediction results of the two models were obtained, as shown in Fig. 14. It can be seen from the Fig. 14 that the weakened semi-analytical solution is in good agreement with the numerical model solution. Thus, it is feasible to adopt the semi-analytical method to conduct actual productivity prediction and dynamic performance analysis for shale gas wells with managed pressure drawdown.

**Table 2:** Well testing interpretation parameters of the numerical model based on the IHS RTA^45^.

Parameters name	Value	Parameters name	Value
Effective Horizontal well length/m	1415	The number of hydraulic fractures	70
Initial formation pressure/MPa	75	the hydraulic fracture half-length/m	100
Formation temperature/K	390	the hydraulic fracture zone half width/m	5
Formation thickness/m	16	well spacing /m	400
Total porosity/%	5.90	Constant bottomhole pressure /MPa	3
Matrix permeability /$(mD)$	0.000456	Rock density/(kg/m^3^)	2500
Dimensionless fracture conductivity	5.0	Langmuir volume/(m^3^/t)	4.0
Fracture permeability/$(mD)$	0.0117	Langmuir pressure /MPa	8

**Figure 15:**
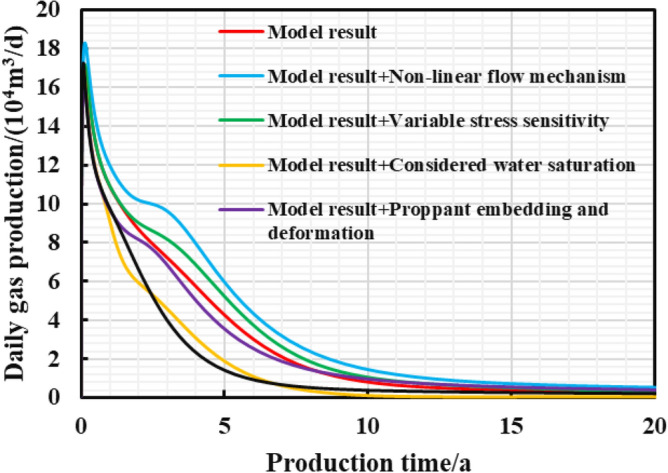
Diagram of the cumulative gas production and time for different models.

**Figure 16:**
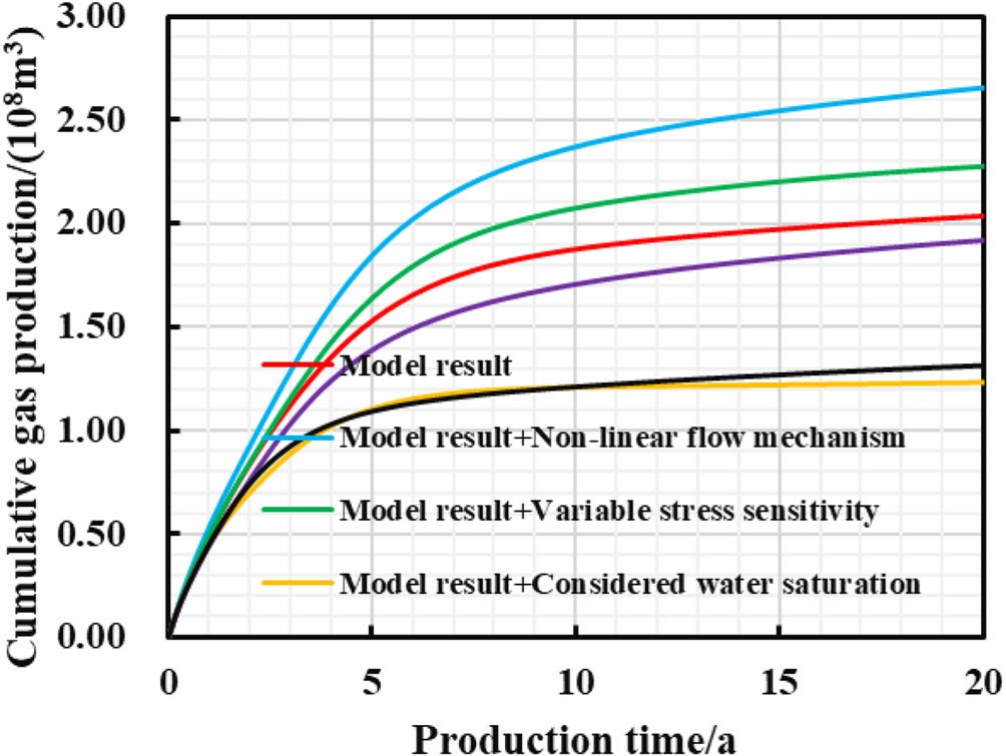
Diagram of the daily gas production and time for different models.

**Figure 17:**
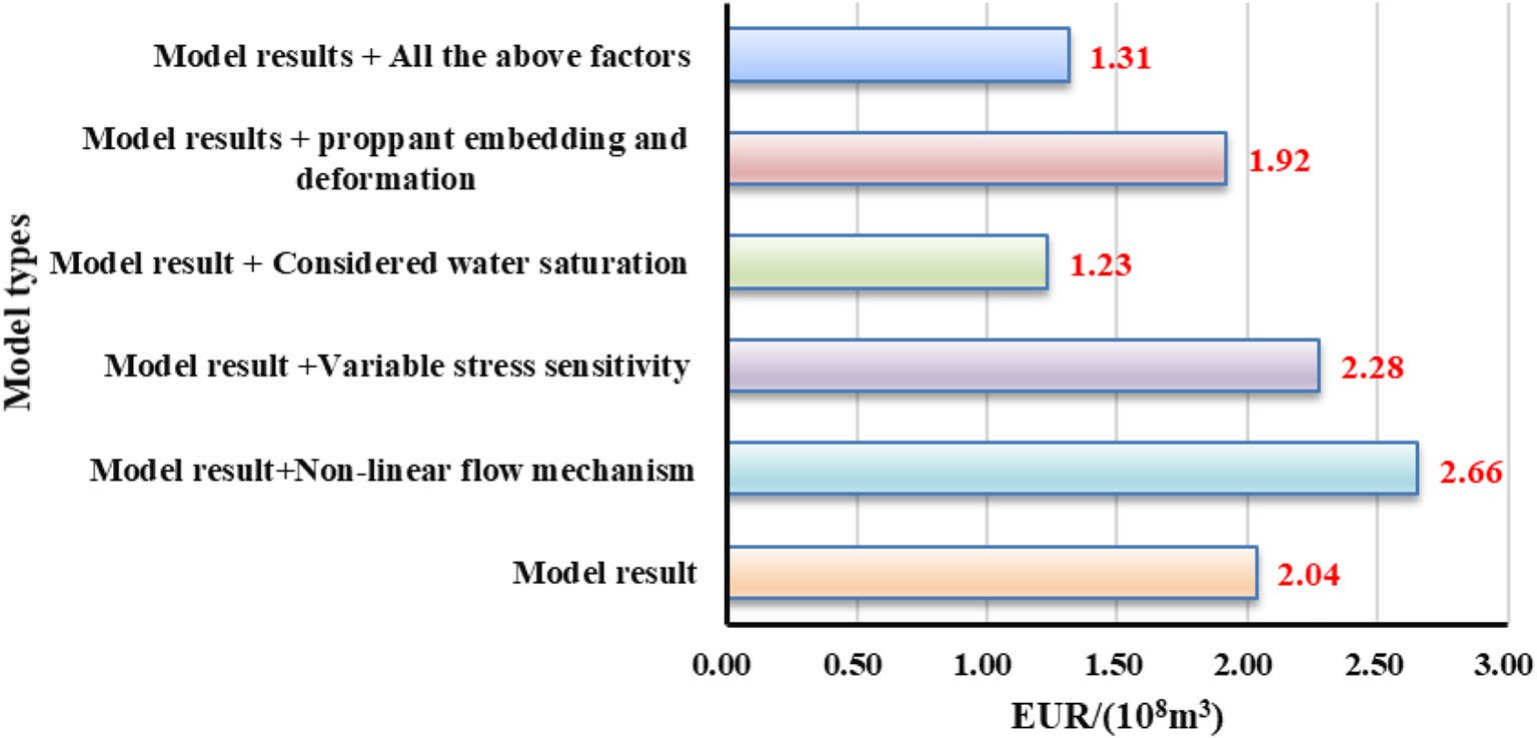
Diagram of the EUR comparison for different models.

Figures 15, 16, 17 show that when the non-linear gas flow mechanism is not considered in the production model, the peak daily gas production is low, and the daily gas production decline significantly. The production capacity predicted by the model can be underestimated by 30.3%, indicating diffusion and desorption is significant to the later-staged-productivity; the calculated EUR of considering variable stress sensitivity is 227.7 million cubic meters, 11.8% higher than that calculated by the constant stress sensitivity productivity model. The increase in net stress leads to the reservoir compaction, and the reduced reservoir seepage capacity; the model considering the reservoir water saturation of 45% predicts that EUR is 39.4% lower than that of dry reservoir. It can be seen that the fracturing fluid indeed caused damage to reservoir seepage capacity; when the gas well is put into production, the hydraulic fracture proppant can suffer elastic embedding and elastic deformation to result in 5.75% loss of the EUR. If the above comprehensive factors are considered in the model, the final EUR is about 35.5% lower than that of the weakened model. To sum up, the comprehensive factors considered in the production model is conducive to improve the accuracy of predicting the medium and long-term productivity. It aims to provide theoretical reference and engineering value for the optimization analysis of pressure reduction strategies.

## Case study

### Production effect analysis between managed drawdown and high drawdown

**Figure 18:**
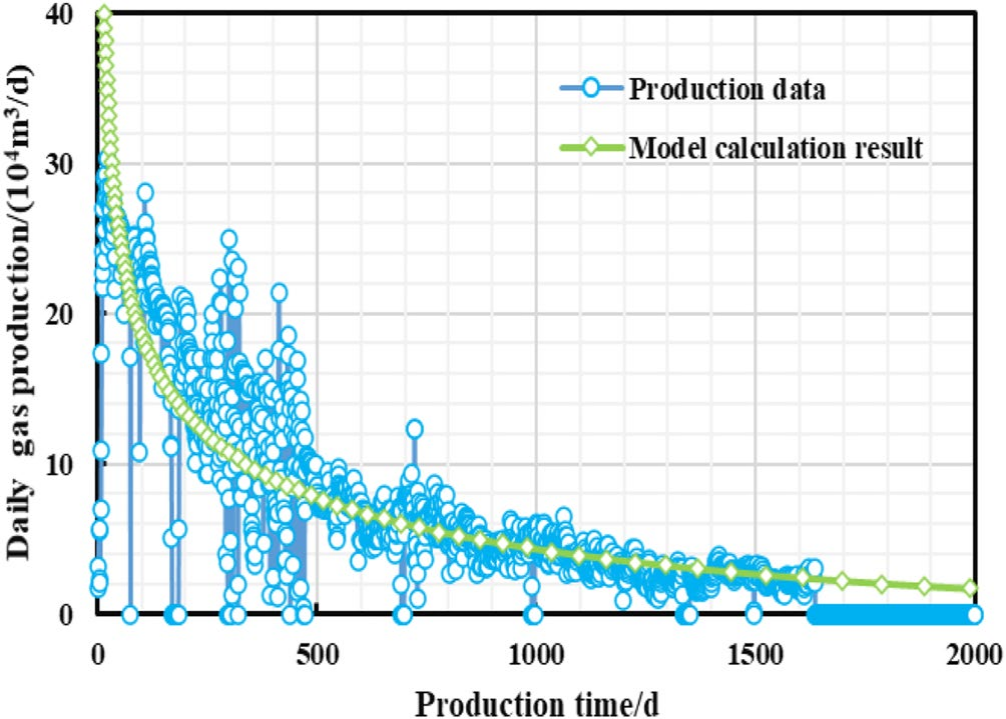
Production effect fitting of HD well.

**Figure 19:**
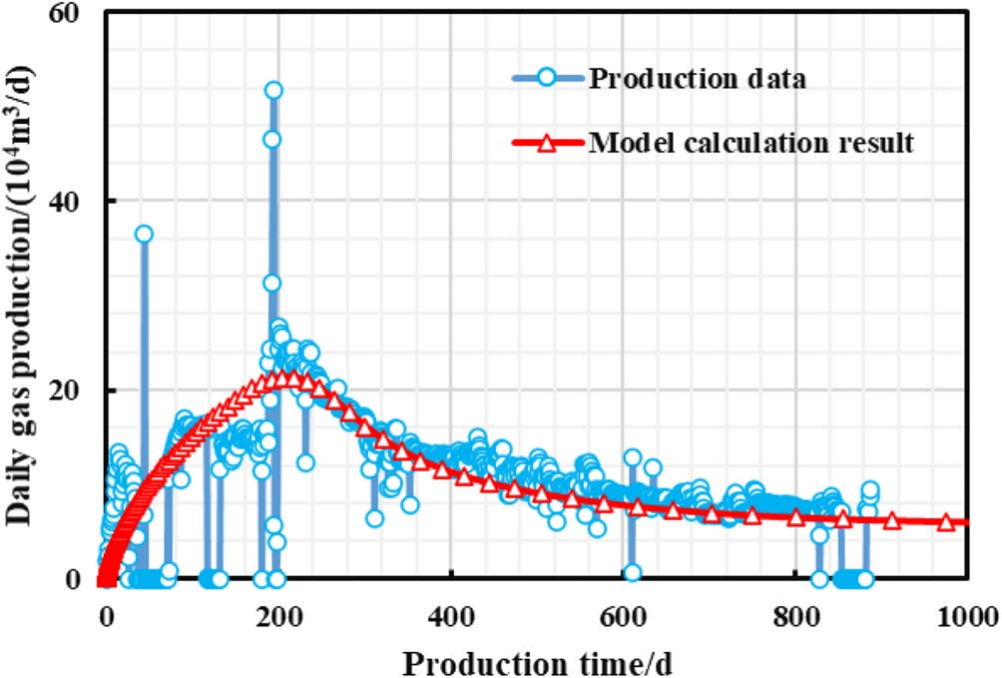
Production effect fitting of HD well.

**Figure 20:**
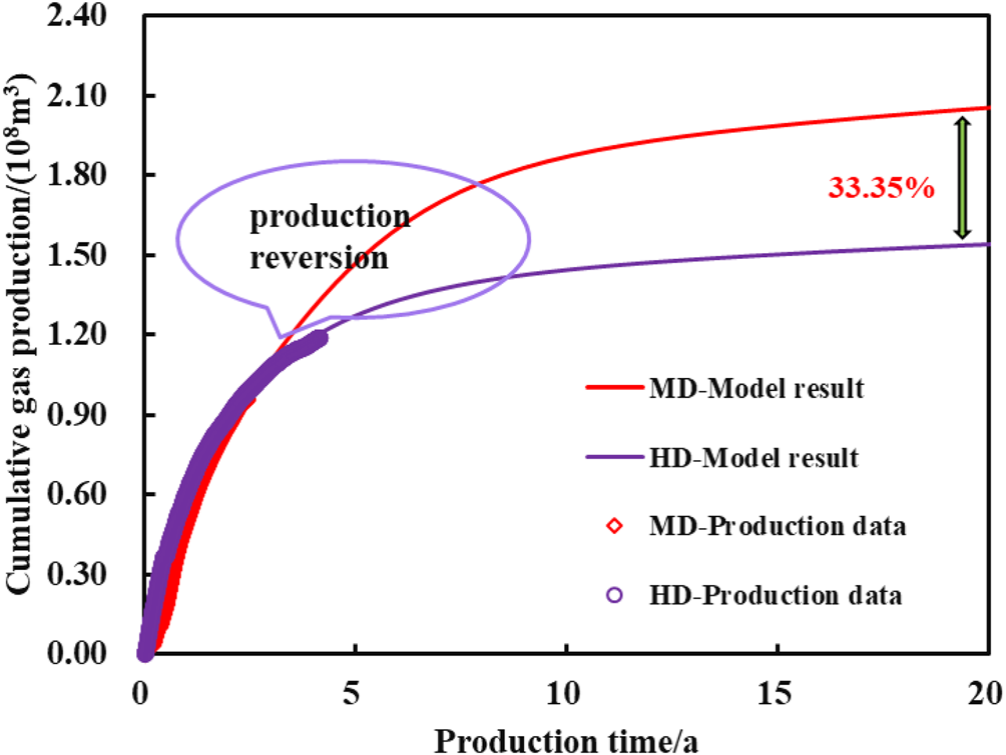
Prediction and comparison of the production effect of MD and HD.

Two shale gas wells with different production systems, HD (High Drawdown) and MD (Managed Drawdown), were selected as production effects comparison for basically same testing production, the number of fracturing sections, and geological background. HD well adopts short-term pressure control method for production, while long-term pressure control strategy is applied to MD well. The constant pressure productivity model was used to historically fit the production data of the HD well (Fig. 18) and the pressure-controlled productivity model in the paper was used to historically fit the MD gas well production data (Fig. 19) to invert the unknown geological and engineering parameters of the two wells, and then respectively forecast the EUR for two wells. As is shown in Fig. 20, the short-term-cumulative gas production of managed pressure drawdown is lower than that of high pressure drawdown. The advantages of managed drawdown production in the later stage begin to appear, and "capacity reversal" occurs. The ultimate EUR calculated by the proposed model is about 33.35% higher than that of the constant pressure productivity model. Therefore, the managed pressure drawdown production system can achieve the production goal of increasing the medium and long-term cumulative production of a single well.

**Figure 21:**
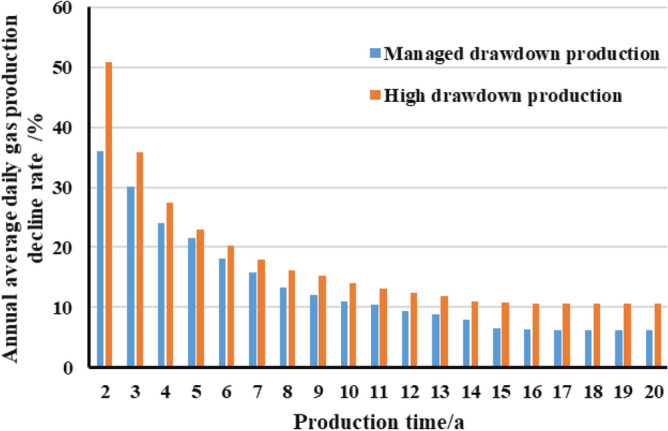
Comparison diagram of the relationship between the daily gas production decline rate and production time of gas wells with MD and HD.

As can be seen in the Fig. 21, the annual average daily gas production decline rate of gas wells with the managed pressre drawdown system is generally lower than that of gas wells with the high pressre drawdown system. Starting from the second year of production, the annual production decline rate of pressure-controlled gas wells has been reduced from 36.02 to 5.12%, and has been relatively low from the 15th year, basically entering the stable production stage. The annual daily production decline rate of depressurized gas wells has been reduced from 50.80 to 10.55%, which is still in a period of obvious decline in production. It can be seen that pressure-controlled production can control the degree of production decline, protect the seepage capacity of the reservoir, extend the stable production period of gas wells, and achieve high-efficiency increase in ultimate production of gas wells.

### Optimization analysis of pressure drawdown management strategy

The pressure drop strategy of an example well in the Sichuan shale gas area was optimized from the production effect and economic benefit evaluation with the proposed productivity model, including the pressure control duration, the initial controlled pressure, and different pressure drop paths, respectively. The geological parameters and engineering of this example well are shown in Table 3.

**Table 3:** Geological parameters and engineering parameters of an example well.

	Parameters name	Value	Parameters name	Value
Basic parameters	Initial pressure/MPa	80	Fractured sections	20
	Reservoir temperature/K	400	Number of fracturing clusters per section	6
	Reservoir thickness/m	16	Constant bottomhole flowing pressure/MPa	3
	Length of horizontal section /m	1800		
Matrix	Matrix permeability/mD	0.0004	Langmuir volume/(m^3^/t)	3.5
	Matrix porosity/%	4.0	Langmuir pressure/MPa	8
	Stress sensitivity coefficient/MPa^-1^	0.20		
Fracture network	Permeability in the fracture network/mD	1	Initial stress sensitivity coefficient/MPa^-1^	0.26
	Porosity/%	5.3		
Hydraulic fracture	Half-length of hydraulic fracture	100	Width of fracturing zone between two clusters /m	10
	Width of hydraulic fracture/m	0.01	Permeability in the hydraulic fracture/mD	50
	Cluster spacing/m	15	Well spacing/m	300

#### The impact of pressure control duration on production effects

**Figure 22:**
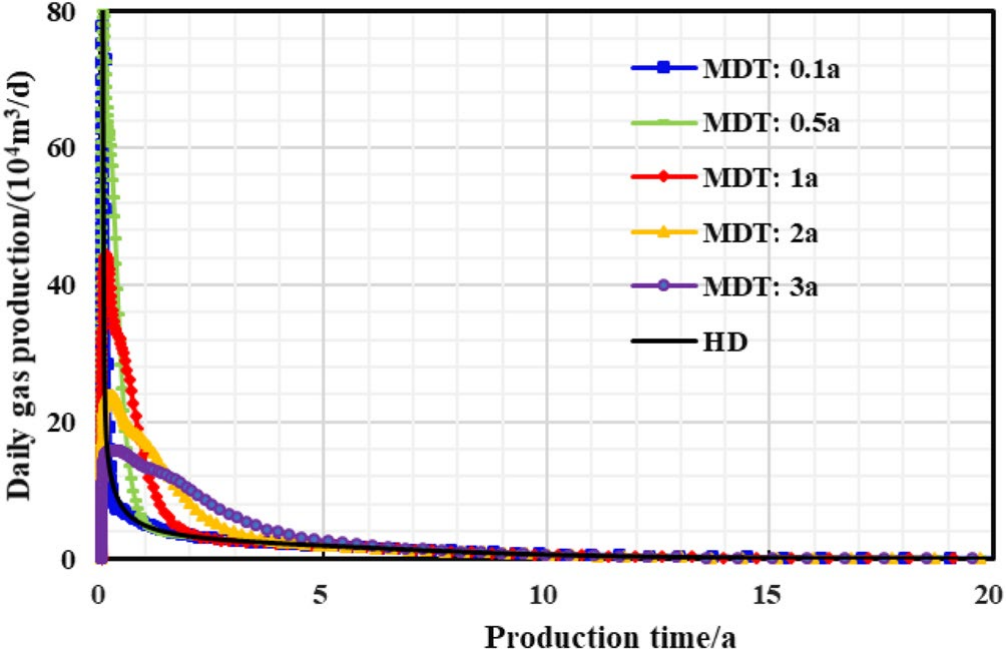
Diagram of daily gas production and time under different pressure control durations.

**Figure 23:**
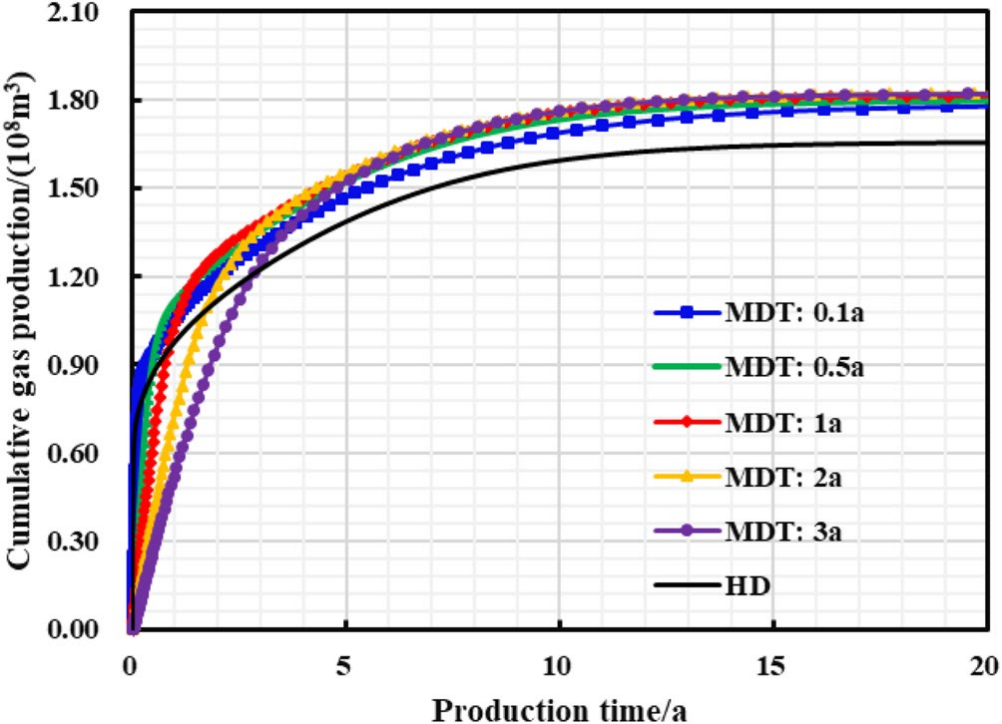
The cumulative gas production and time chart under different pressure control durations.

**Figure 24:**
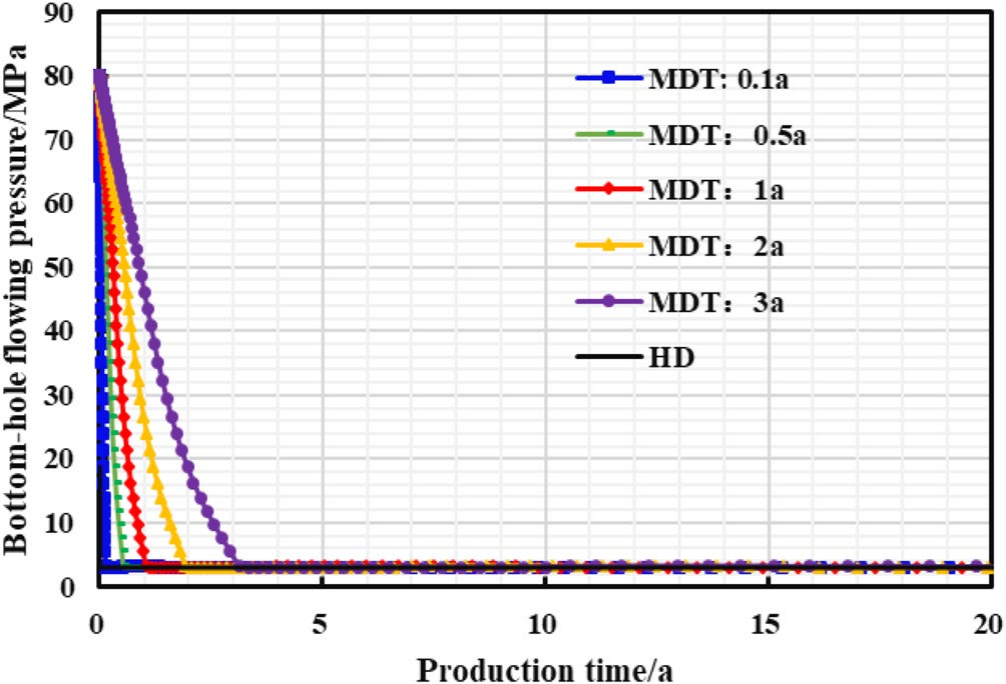
Bottomhole flowing pressure and time chart under different pressure control durations.

**Figure 25:**
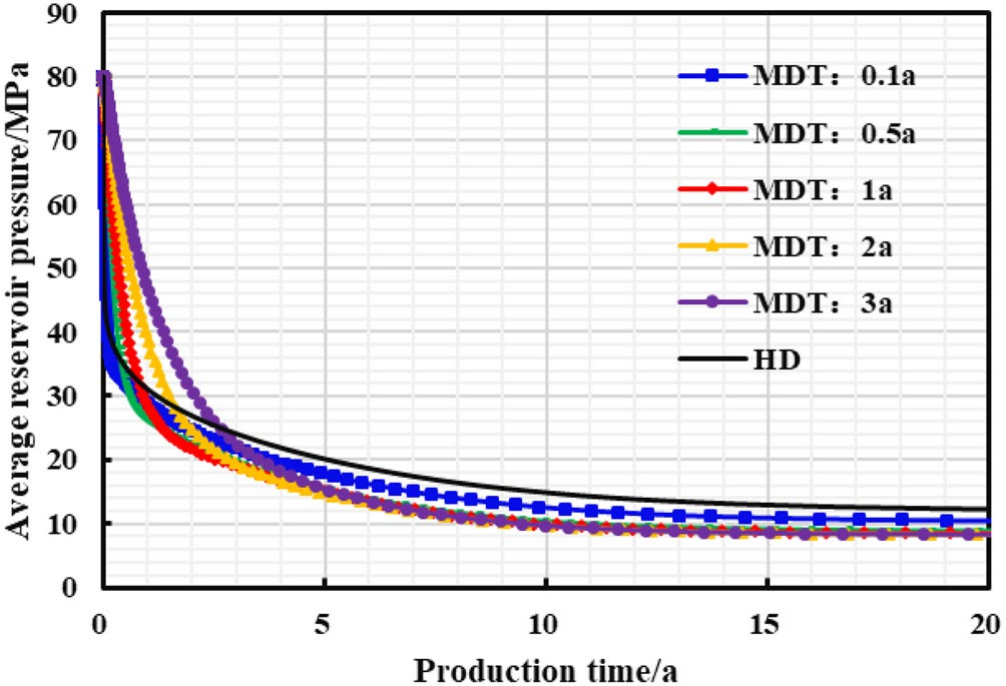
Reservoir average pressure and time chart under different pressure control durations.

**Figure 26:**
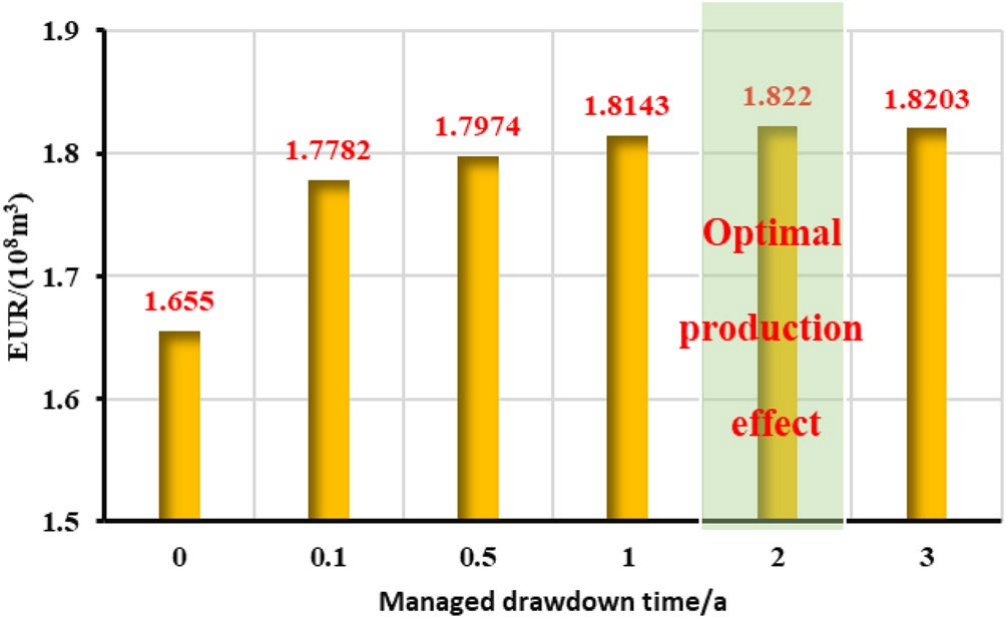
EUR changes under different pressure control durations.

The stepped pressure drop path can be approximated as a linear pressure drop path during the long-term production period of a gas well, thus the limited pressure drop path is linear. The influence of different pressure control durations of the gas well on the production effect was studied, and the optimal pressure control duration was clarified for the example well. As is shown in Figs. 22, 23, 24, 25, 26, comparing the production effect of depressurization production and pressure control for 0.1 to 3 years, with the increase of pressure control duration, the annual average daily gas production of gas wells decreases gradually, and the stable production period lasts for a long time. There is an overall trend of gas well EUR first increasing and then decreasing when the pressure control duration increases. When the pressure is controlled for 3 years, the EUR will show a downward trend, indicating that the pressure control period has an optimal value of 2 years, to obtain the most ideal productivity. Reasonable pressure control production for 2 years can make single EUR be increased by 10.02%. On the contrary, too long pressure control time is not conducive to gas well production performance.

#### The influence of the initial controlled pressure on the production effect

**Figure 27:**
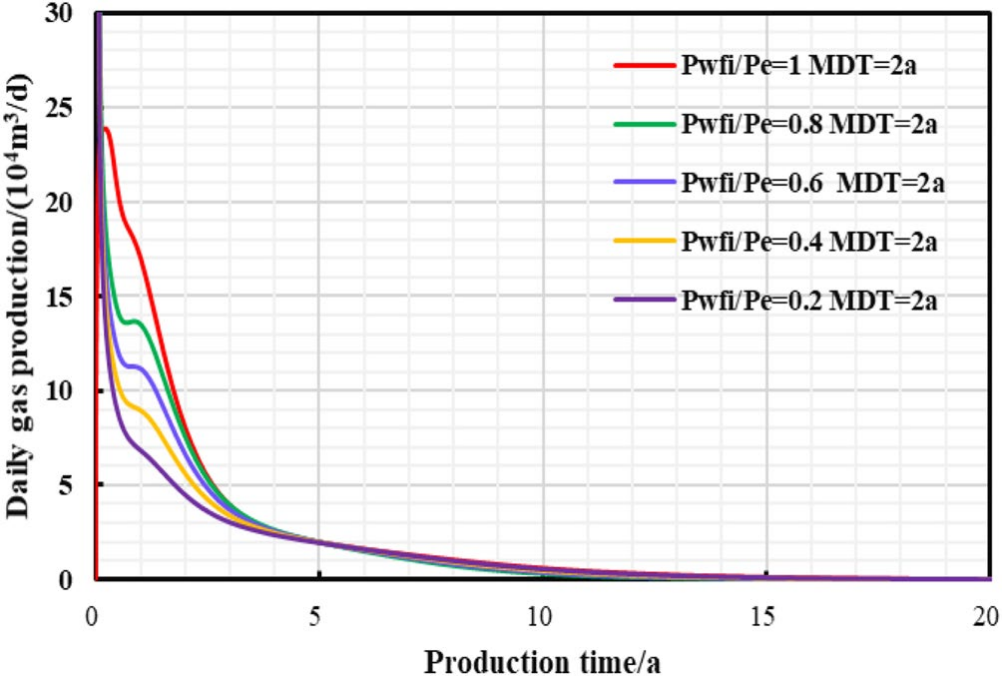
Daily gas production and time chart under different initial controlled pressures.

**Figure 28:**
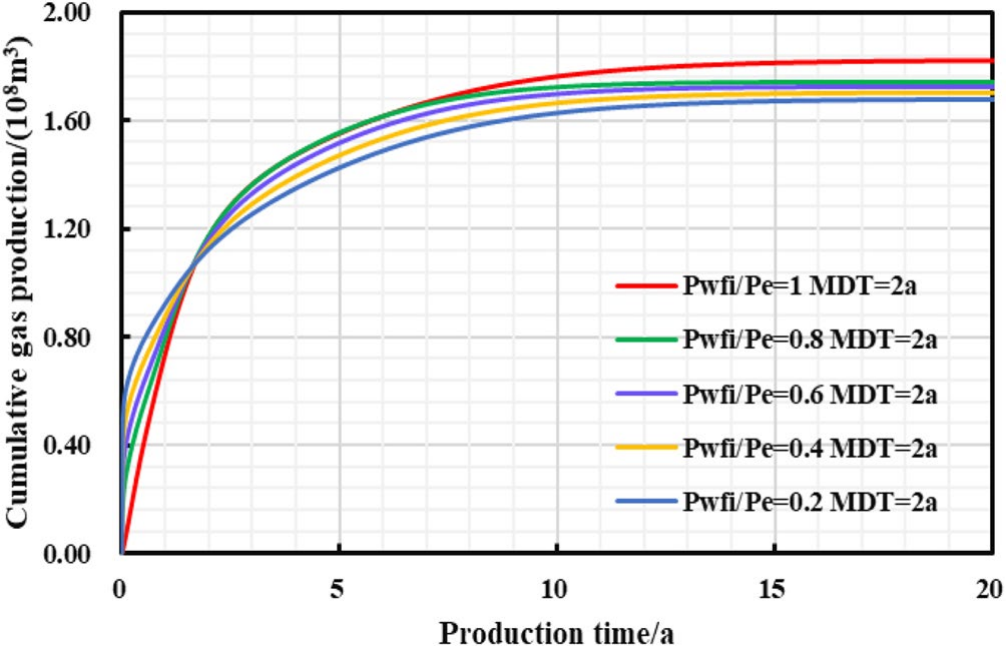
The cumulative gas production and time chart under different initial controlled pressures.

**Figure 29:**
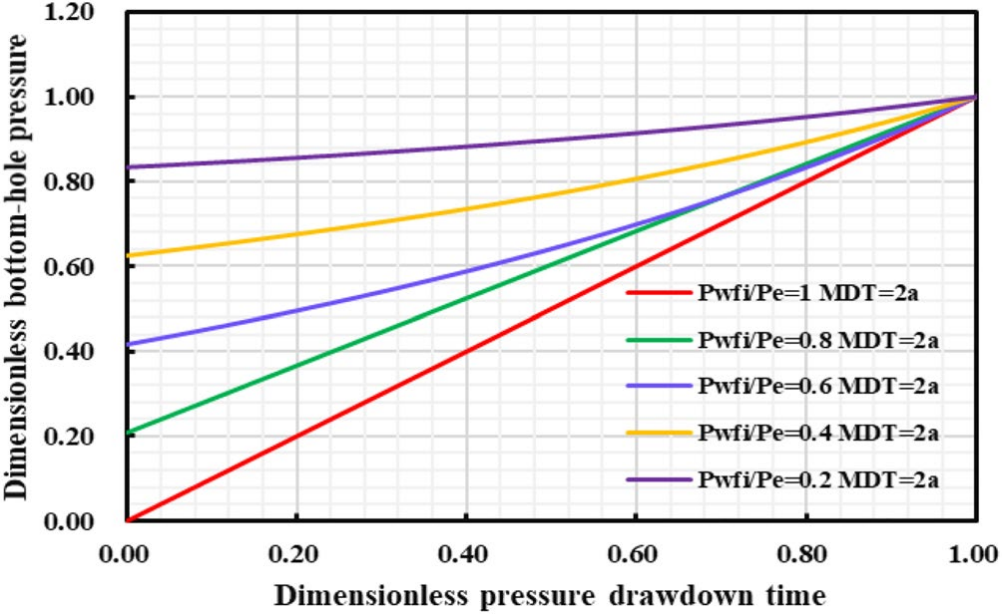
Dimensionless bottom hole and pressure drop time chart under different initial controlled pressures.

**Figure 30:**
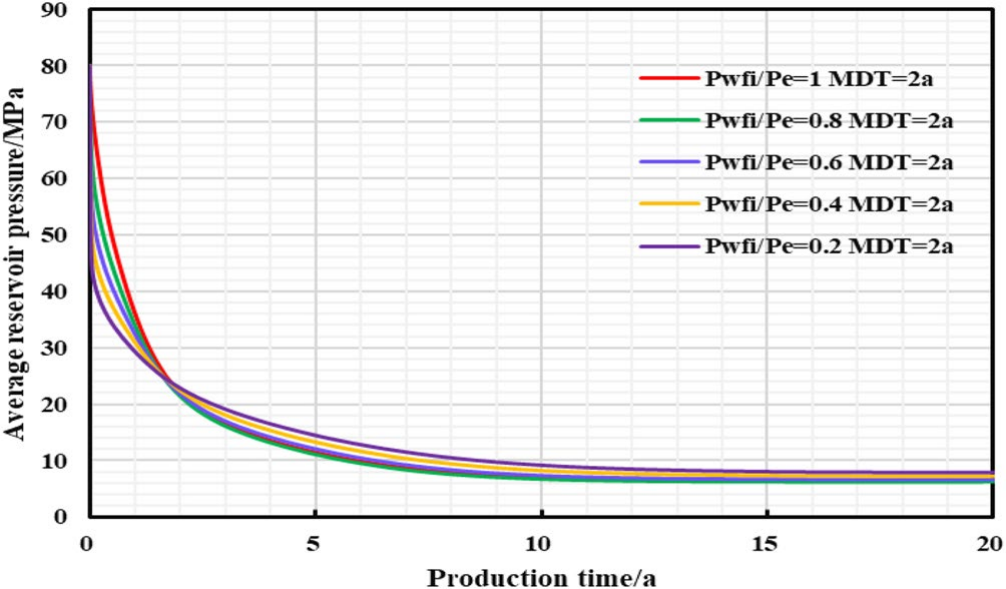
The relationship between average reservoir pressure and time under different initial controlled pressures.

**Figure 31:**
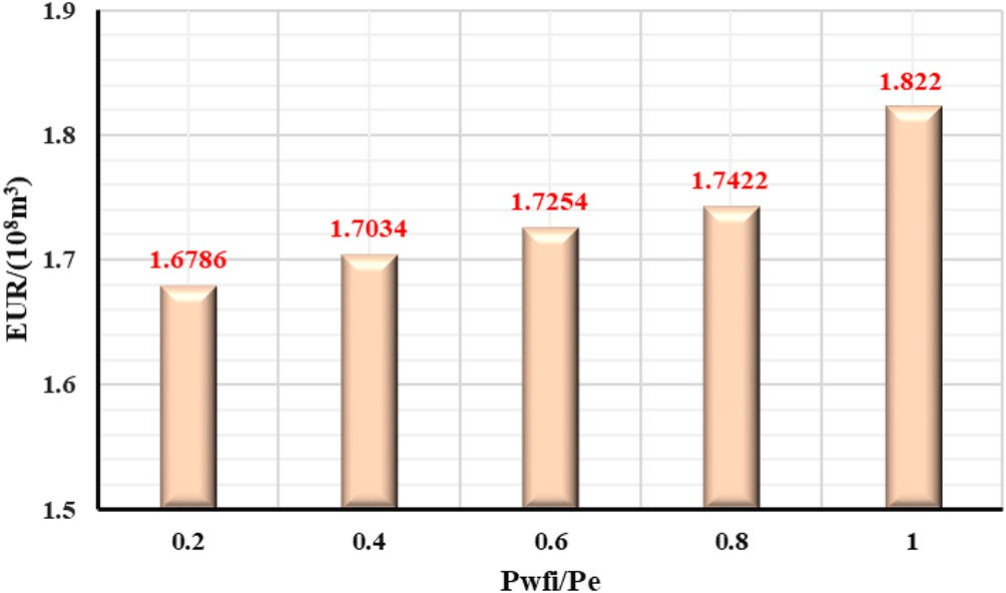
Diagram of the relationship between EUR and the initial controlled pressure.

In the early stage of gas well production, well test work is usually carried out to explain the reservoir properties and parameters, and pressure control will not be implemented from the beginning of production process. Thus, the gas well is set to control pressure from different bottom hole pressures starting point to study the effect of different initial controlled pressures on gas well production. Comparing the production performance charts of gas wells with different initial controlled pressures for 2 years of pressure control (Fig. 27, 28, 29, 30, 31), it can be seen that when the initial controlled pressure is closer to the original reservoir pressure, that is, the pressure is controlled at the beginning of production process for shale gas wells, low annual average daily gas production decline rate and long stable production period can be obtained. Specifically, the EUR of initial reservoir pressure relative to that of 1/5 of initial reservoir pressure be increased by 8.54%. Conversely, the initial controlled pressure is close to delivery pressure, and it is mainly dominated by depressurization production. It can illustrate that the fast pressure drop in the reservoir near the wellbore will lead to high initial gas production of the gas well, and slow reservoir production rate in the later stage of production, which is attributed to permeability loss and thus the low long-term production. Therefore, the formation pressure drops slowly when the pressure is controlled in the early stage of production, and the long-term cumulative gas production of gas wells is greater, on the contrary, the cumulative gas production of depressurization is the lowest.

#### Influence of pressure drop path type on production effect

**Figure 32:**
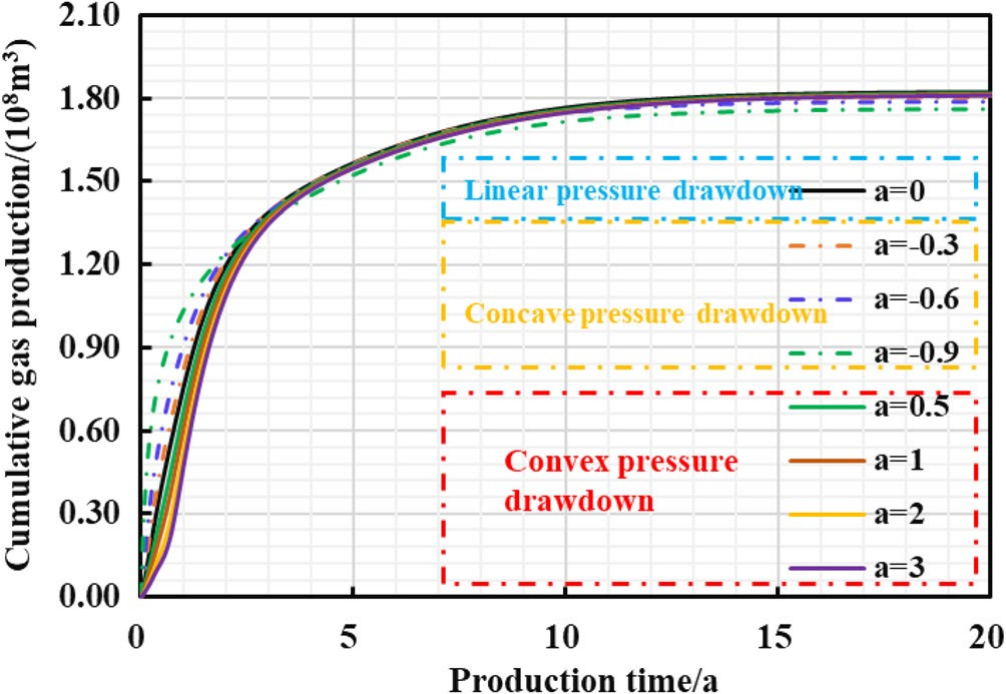
The cumulative gas production and time chart under different pressure drop paths.

**Figure 33:**
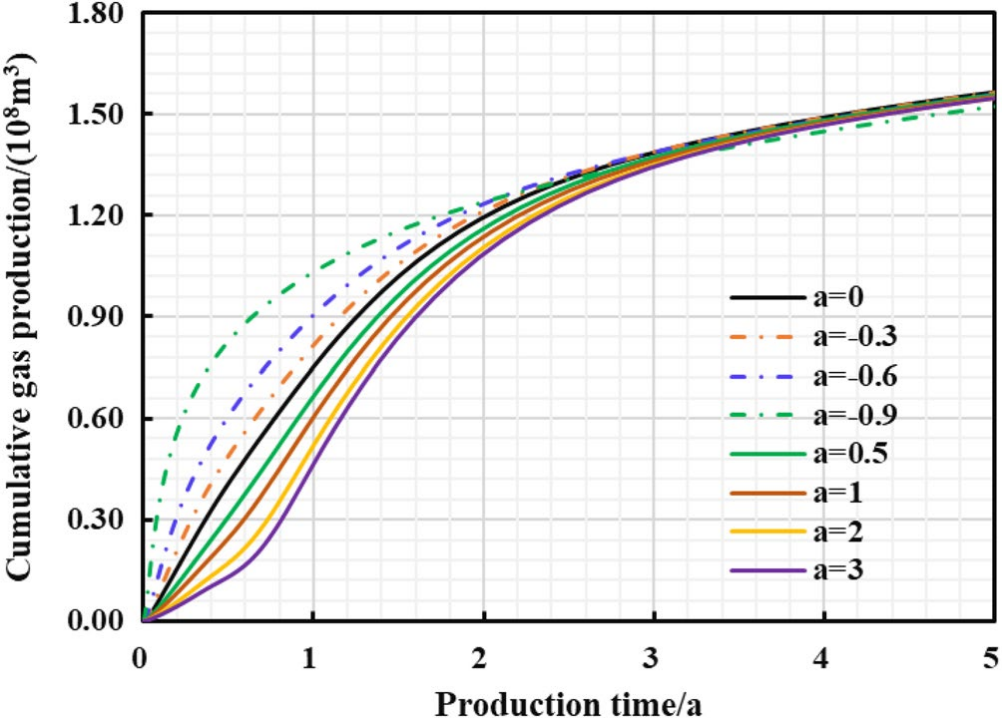
The cumulative gas production and time chart in the early stage of production.

**Figure 34:**
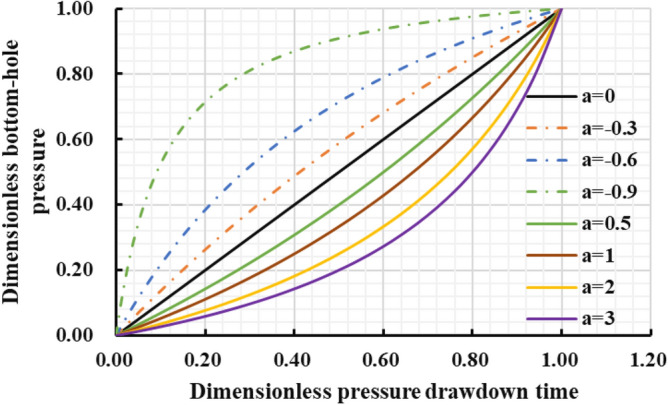
Dimensionless bottom hole pressure and pressure drop time chart under different pressure drop paths.

**Figure 35:**
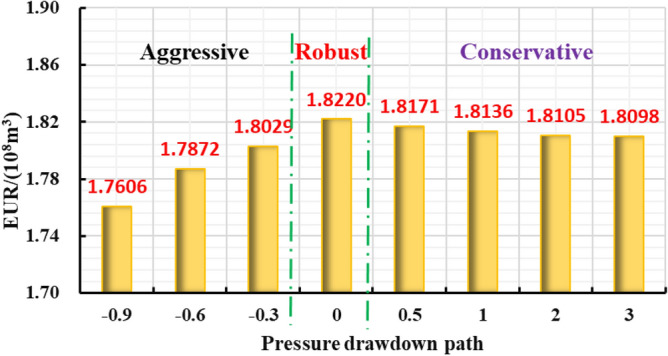
Diagram of relationship between EUR and pressure drop path.

Different reservoir stress sensitivity at different pressure drop paths can lead to obvious distinction in reservoir seepage capacity, which affects the final dynamic production performance. Thus, the production effect of gas well under different pressure drop paths was evaluated (Fig. 32, 33, 34, 35): The production effect of a single well is the most ideal when bottom hole flowing pressure decreases linearly, that is, a robust drawdown. When the pressure drop occurs as a conservative drawdown, too low pressure drop rate is not conducive to increasing the long-term production of gas reserves in the matrix. On the contrary, the prominent concave effect of pressure drop path means great production pressure difference near the well, and can cause serious damage to the fractured reservoir conductivity and the boundary production utilization of reservoir reserves.

#### The influence of managed pressure drawdown parameters on economic benefits

The final objective function for optimizing the economic benefits of shale pressure controlled gas wells is determined by maximizing cumulative gas production and minimizing production costs, that is, the 20-year net present value (NPV), and an economic evaluation model is introduced^46^:

43 $$NPV = \sum\limits_{j = 1}^{n} {\frac{{m(G_{p,j} - G_{p,j - 1} )}}{{(1 + i_{r} )^{j} }} - Q_{0} }$$

where $i_{r}$ is annual interest rate,%; $m$ is gas price, yuan/m^3^.

Assuming that the investment cost of a single horizontal well in this example is 68 million yuan, the gas price is 1.275 yuan/m^3^, and the annual interest rate is 10%, study the impact of different pressure control durations, pressure drop paths, and initial controlled pressures on net present value to seek the optimal combination of pressure drop parameters and obtain the greatest economic return, and formulate the best economic pressure control plan.

**Figure 36:**
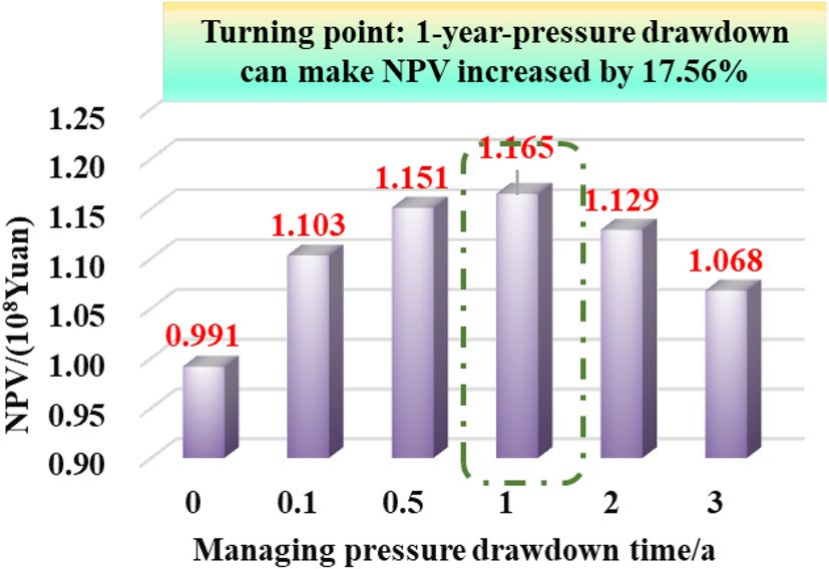
20-year NPV chart for different pressure control duration.

**Figure 37:**
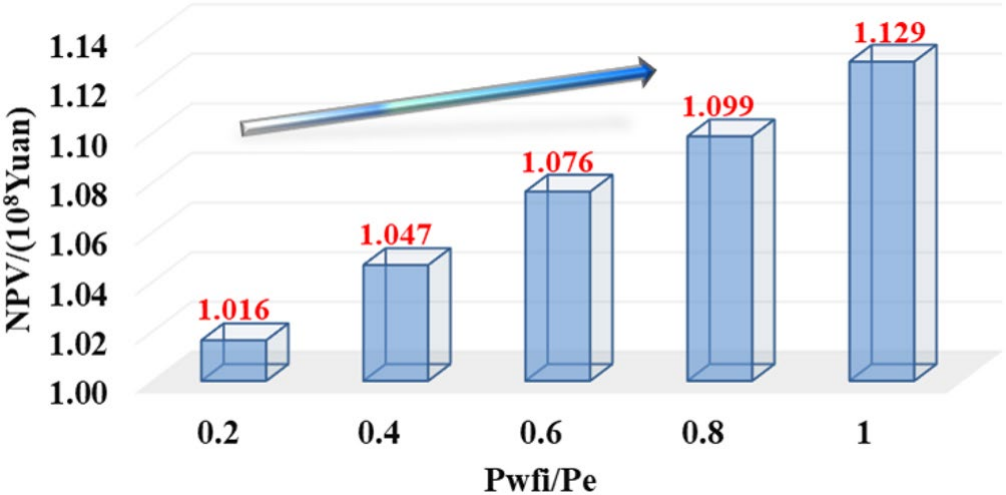
NPV chart of different initial controlled pressures.

**Figure 38:**
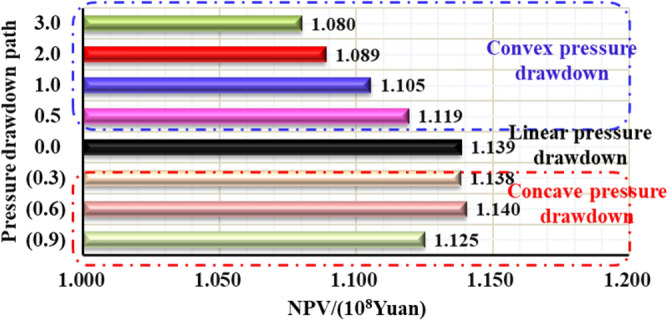
20-year NPV chart under different pressure drop paths.

Comparing the NPV chart of 0.1~3-year-pressure controlled production and depressurization production (Fig. 36), the ultimate NPV value of 1-year-managed pressure production is the highest, 17.56% higher than that of depressurization production; Fig. 37 reveals the 20-year NPV value is positively correlated with the initial controlled pressure. The max NPV value can be up to 11.12%; As is shown in Fig. 38, the simulated deep gas well is produced with different pressure drop paths from commissioning to abandonment (20 years of production). The 20-year-NPV of linear pressure drop path is better than the conservative path and the aggressive path. , Which can be increased by 5.46% and 1.24% respectively.

## Summary and conclusions

1. The importance of water–rock interaction of the unproppant fractures to reservoir seepage capacity and long-term production was emphasized in pressure control mechanism. A five-zone composite pressure control productivity model was proposed with comprehensively considering the multiple pressure control mechanisms, and then verified to be reliable with numerical simulator. Then it was proved that more comprehensive gas flow mechanisms and pressure control mechanisms considered in the proposed model can effectively promote higher prediction accuracy in the medium and long-term productivity of the gas well, which aims to provide theoretical basis for the optimization analysis of pressure drop strategy.
2. The production effect between the managed pressure drawdown and high pressure drawdown was compared and analyzed by the proposed model. It was found that the annual average daily gas production decline rate by managed drawdown production was generally lower than that of high drawdown production.Theshale gas well controlled by managed drawdown scheme has a relatively earlier stable-production stage; Compared to depressurization production, the production reversion can occur in the controlled pressure production process and the EUR of single well can be increased by about 30%. Thus, for strong-sensitive-shale formations, pressure-controlled production can alleviate the production decline rate, and finally achieve the production goal of increasing the medium and long-term cumulative production.
3. The optimization analysis of pressure drawdown strategy on productivity was carried out.There is an optimal value for the pressure control time of a specific gas well. In this work, two-year-pressure control production can increase 10.02% of the single EUR. On the contrary, too long pressure control time is not conducive to gas well production; when pressure control begins as soon as a gas well is put into production, the productivity can reach the peak and thus the EUR can be increased by 8.54% compared with the depressurization production. The aggressive or conservative bottom hole pressure drop path is not conducive to the long-term production of a single well, while the steady pressure drop path has the least conductivity loss of gas wells to obtain the most ideal reservoir utilization.
4. The economic evaluation model was introduced to formulate the optimal economic pressure drawdown strategy. Generally speaking, the best economic solution can generally be obtained in the initial stage of production.The optimal economic pressure control duration is at an inflection point. The economic benefits first increase and then decrease with the increase in the pressure control duration; Furthermore, the 20-year-NPV value of gas wells is positively correlated with the initial controlled pressure; The NPV of the gas well with the linear pressure drop path is better than the conservative path and the aggressive path. To sum up, the research results can be closely linked to the on-site production practice of shale gas wells, and is of theoretical reference significance and engineering application value for formulating the optimized production strategy plan to improve the ultimate recovery rate of a single well.

## Data Availability

The datasets generated and/or analyzed during the current study are not publicly available due to the fact that the data forms part of current ongoing project but are available from the corresponding author on reasonable request.
